# A Review of Chitosan-Based Electrospun Nanofibers for Food Packaging: From Fabrication to Function and Modeling Insights

**DOI:** 10.3390/nano15161274

**Published:** 2025-08-18

**Authors:** Ji Yang, Haoyu Wang, Lihua Lou, Zhaoxu Meng

**Affiliations:** Department of Mechanical Engineering, Clemson University, Clemson, SC 29634, USA

**Keywords:** chitosan (CS)-based electrospun nanofibers, food packaging, polymer blending, inorganic additives, computational modeling and simulations, challenges and future perspectives

## Abstract

Food is fundamental to human survival, health, culture, and well-being. In response to the increasing demand for sustainable food preservation, chitosan (CS)-based electrospun nanofibers have emerged as promising materials due to their biodegradability, biocompatibility, and inherent antimicrobial properties. When combined with other biopolymers or bioactive compounds, CS-based nanofibers offer enhanced functionality for applications in food packaging, preservation, and additives. This review summarizes recent advances in the fabrication and performance of CS-polymer and CS-inorganic composite nanofibers, with a focus on their mechanical strength, thermal stability, barrier properties, and antimicrobial efficacy. The use of these nanofibers across a range of food categories—including vegetables, fruits, fresh-cut produce, dairy products, meat, seafood, and nuts—is examined. Beyond experimental approaches, the review also explores the growing role of computational simulations in predicting the mechanical strength, barrier performance, antimicrobial activity, and biodegradability of CS-based nanofibers. Key modeling techniques and simulation tools are summarized. Finally, current challenges and future research directions are discussed, underscoring the potential of CS-based electrospun nanofibers as sustainable and multifunctional solutions for modern food packaging. By integrating experimental advancements with computational insights, this review provides a comprehensive and forward-looking perspective on CS-based electrospun nanofibers for food packaging.

## 1. Introduction

Food is a fundamental pillar of human survival, health, culture, and well-being [1]. The modern food system encompasses a wide range of interconnected processes, including food production, processing, preservation, distribution, marketing, purchasing, consumption, and waste management [2]. Despite tremendous efforts, the Food and Agriculture Organization of the United Nations reported that approximately one-third of global food production, totaling ~1.3 billion tons, was wasted without being consumed in 2021 [3]. This issue is particularly acute for perishable foods such as vegetables, fruits, meats, and seafood, which are highly susceptible to spoilage and degradation during storage and handling [4]. Microbial contamination and biochemical deterioration reduce freshness and render such products unfit for consumption, contributing significantly to food waste [5].

In response to the growing demand for fresh, high-quality food with extended shelf life, researchers are actively exploring advanced preservation technologies. Among them, membrane-based preservation has gained prominence, particularly nanofiber-based membranes, due to their exceptional functional and barrier properties. These membranes offer enhanced antimicrobial activity, oxidation resistance, and improved gas and water vapor barrier performance [6,7]. As a result, nanofiber-based membranes can effectively maintain the nutritional and sensory qualities of perishable foods—including fruits, vegetables, dairy, meat, and seafood—while inhibiting microbial growth and extending shelf life [8].

Several fabrication methods have been employed to produce nanofibers, ranging from high-throughput techniques such as melt fibrillation, island-in-sea spinning, and gas jet spinning to highly controlled approaches like nanolithography and molecular self-assembly [9,10,11,12,13,14]. However, many of these techniques are limited by factors such as restricted material compatibility, complex fiber alignment requirements, high cost, and low production efficiency [10,15]. In contrast, electrospinning has emerged as a widely used and versatile technique for nanofiber fabrication. It enables the production of continuous fibers with diameters in the micro- to nanometer range through a relatively simple setup [16,17].

Importantly, electrospinning offers key advantages over traditional encapsulation methods such as spray-drying or heat-drying, particularly for temperature-sensitive compounds, as it operates under ambient or low-temperature conditions [18]. The process involves applying a high-voltage electric field to a polymer solution, resulting in the formation of a charged jet that elongates and solidifies into fibers upon solvent evaporation. The resulting electrospun nanofibers possess a high specific surface area, tunable porosity, and favorable physicochemical characteristics for encapsulating and releasing bioactive compounds [19,20,21].

Chitosan (CS) is a widely utilized material for food preservation, food additives, and active packaging. Derived from chitin, a naturally abundant polysaccharide found in the exoskeletons of crustaceans such as crabs and prawns, CS offers significant advantages for food preservation applications, thanks to its film-forming ability and additional antioxidant, anti-inflammatory, and antimicrobial effects [22]. Furthermore, its sustainable production from chitin ensures scalability for industrial applications [23]. Global Warming Potential (GWP) is a widely used metric for quantifying sustainability. For example, Ponnusamy and Mani [24] performed a life cycle assessment (LCA) on CS-based biopolymer films using GWP as an impact metric. Their analysis showed that the GWP of manufacturing CS-based films was approximately 3.91 kg CO_2_ eq./kg, which is notably lower than that of typical fossil-based polymer films. Furthermore, scenario analysis demonstrated that incorporating cellulose nanofibrils (CNF) not only improved the mechanical and barrier properties of the films but also did not increase the overall GWP. The cradle-to-gate LCA results confirm that CS-CNF composite films have a reduced environmental footprint compared to conventional petroleum-based alternatives, providing quantitative support for the sustainability of CS-based materials in food packaging applications. In recent years, CS-based nanofibers have garnered considerable attention due to their superior functionalities, including biodegradability, biocompatibility, barrier performance, and antibacterial activity [25]. Numerous review articles have explored CS-based nanofibers for a wide range of applications, including biomedical uses [26,27,28,29,30,31,32], carriers of bioactive food ingredients [33,34], dye removal [35,36,37], biosensors [38], and food industry applications [16,23,39,40,41]. These reviews typically focus on electrospinning techniques, spinnable materials, and their general applications in the food industry.

However, there remains a critical gap as no existing review comprehensively addresses CS-based electrospun nanofibers incorporating natural polymers or inorganic components, their integration methods, their specific roles across different food types, and critically, the molecular mechanisms underlying their functionality as revealed by computational modeling and simulations.

Computational modeling and simulations play a pivotal role in elucidating the molecular mechanisms responsible for food preservation. Simulation techniques such as Density Functional Theory (DFT) [42], Monte Carlo (MC) simulations [43], and Molecular Dynamics (MD) simulations [44] offer valuable insights into the physicochemical behaviors of CS-based systems. Among these, MD simulations have been most extensively employed to investigate the molecular and atomic-level structure-property relationships of CS and CS-based materials [45]. For example, the review paper by Gade [46] explores a variety of MD approaches—ranging from all-atomistic to coarse-grained (CG) simulations—highlighting their importance in analyzing the structural dynamics and interfacial interactions of CS-based nanocomposites. Michna and colleagues [47] reviewed the combination of modeling and experimental approaches to characterize the conformational properties of CS molecules, using MD simulations to quantify features such as contour length and chain diameter.

Despite these advances and comprehensive reviews, there is currently no dedicated review focusing on CS-based electrospun nanofibers for food packaging that integrates both experimental findings and computational simulations. This review aims to fill that gap by offering a comprehensive overview of CS-based electrospun nanofibers from both experimental and molecular modeling perspectives. We will begin by outlining the intrinsic properties of CS and key challenges, such as poor solubility, high viscosity, and sensitivity to molecular characteristics, alongside strategies like polymer blending, solvent optimization, and crosslinking to improve electrospinnability and fiber quality. We then examine the structural and functional enhancements achieved through CS-polymer and CS-inorganic nanocomposites. Afterwards, we discuss their functional properties—mechanical strength, moisture resistance, antioxidant capacity, and antimicrobial activity—in the context of food applications. Specific uses in the packaging and preservation of vegetables, fruits, dairy, meat, seafood, and nuts are explored. Next, we summarize recent progress in MD simulations related to CS-based nanofibers, highlighting their role in elucidating structure-function relationships. Finally, we address current challenges and propose future research directions to advance the development of sustainable, high-performance CS-based materials for the food industry.

By bridging experimental data with computational insights, this review offers researchers and practitioners a holistic and forward-looking perspective on the design and optimization of CS-based electrospun nanofibers for food packaging applications.

## 2. Emerging Components and Advanced Incorporation Methods

### 2.1. Advances and Challenges in Electrospinning CS

CS, derived from crustacean exoskeletons and fungal cell walls [48,49], has gained widespread attention for food packaging due to its biocompatibility, biodegradability, and antimicrobial properties. Its cationic nature enables interactions with negatively charged microbial membranes, disrupting cell integrity and thereby extending the shelf life of perishable foods [50,51]. CS also forms flexible, transparent films with good mechanical and oxygen barrier properties when processed via electrospinning [52,53]. These features make CS a highly suitable matrix for developing active, biodegradable packaging materials.

**Figure 1:**
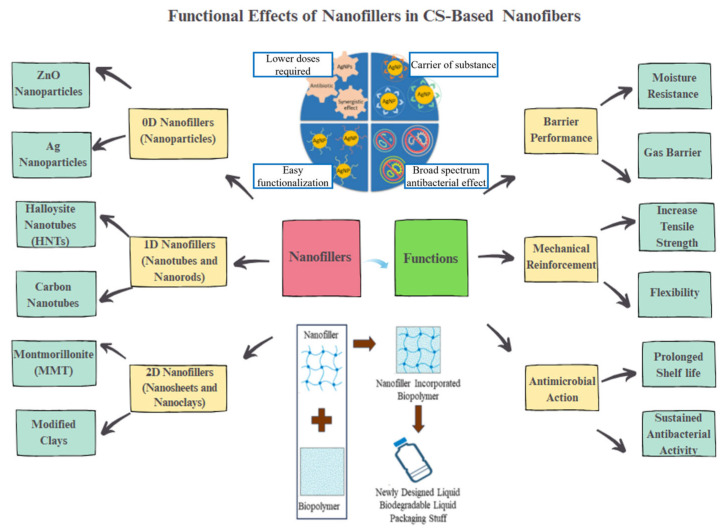
Schematic illustration of various nanofillers and functions of CS-based nanofibers. Reprinted with permission from refs. [55,56]. Copyright Elsevier, 2024; MDPI, 2021.

Electrospun CS nanofibers have a high surface area and tunable porosity, which facilitates the incorporation of functional additives, including bioactive agents and nanofillers [54]. Nanofillers can be categorized by dimensionality: 0D (e.g., ZnO and Ag nanoparticles), 1D (e.g., halloysite nanotubes, carbon nanotubes), and 2D (e.g., montmorillonite, modified clays). These fillers improve CS-based nanofibers by enhancing gas/water barrier properties, mechanical strength, and antimicrobial efficacy, as summarized in Figure 1. Despite these functional benefits, their incorporation raises food safety and edibility concerns, especially in direct-contact applications.

In contrast, essential oils (EOs)—such as oregano, thyme, and cinnamon oils—are recognized as safe, edible additives with strong antimicrobial and antioxidant activities [57]. EOs can be co-loaded with nanofillers into CS-based nanofibers to achieve synergistic effects and sustained release. For instance, montmorillonite and halloysite nanotubes can serve as reservoirs for hydrophobic, volatile EO compounds, allowing for their controlled encapsulation and sustained release [58]. Zhan et al. [59] demonstrated that an EO-loaded CS/Zn^2^-modified montmorillonite (Zn-MMT) system exhibited a remarkable EO loading capacity and superior antibacterial efficacy. These materials retain the edibility of CS and EOs, although the overall safety still hinges on the food-grade quality of the incorporated nanomaterials.

Despite the huge potential and versatility of CS-based electrospun nanofibers, electrospinning CS presents several challenges [60,61,62]. The molecular weight (MW) and degree of deacetylation (DD) of CS significantly influence its nanofiber formation behavior. Higher MW CS (310–375 kDa) tends to produce fiber diameters larger than 100 nm due to increased solution viscosity, while lower MW CS (50–190 kDa) may result in bead formation or discontinuous fibers [63,64]. DD affects the charge density and solubility of CS, thereby impacting the solution’s conductivity and spinnability. Studies have shown that CS, with a MW of ~106,000 g/mol and a concentration of 7–7.5%, can generate consistent and uninterrupted fibers [65]. Meanwhile, CS’s polycationic nature, high viscosity, and strong intermolecular hydrogen bonding hinder the formation of continuous and uniform fibers.

CS’s limited solubility in common organic solvents further complicates processing [23,30,48]. It is typically dissolved in acidic systems such as acetic, formic, lactic, or citric acid, or in specialized mixed systems such as trifluoroacetic acid (TFA). Additionally, the concentration and type of solvent (e.g., acetic, formic, and trifluoroacetic acids) play a critical role in enhancing the solubility and electrospinnability of CS [66,67]. Among various solvent systems explored, the TFA/dichloromethane (DCM) mixture has been identified as one of the most effective for electrospinning CS. This is attributed to its favorable influence on key solution properties, including conductivity, surface tension, and viscosity, all of which directly impact fiber formation. Moreover, fine-tuning other electrospinning parameters, such as applied voltage, flow rate, tip-to-collector distance, and ambient conditions (temperature and humidity), is crucial in achieving uniform fiber morphology [68,69], as summarized in Table 1. 15–25 kV and 0.2–1.0 mL/h is the typical range of voltage and flow rate for electrospinning CS.

To further enhance the functional performance of CS fibers, advanced electrospinning techniques such as coaxial and emulsion electrospinning have been explored. These methods enable the fabrication of core–shell structures that encapsulate active agents [80,81]. Coaxial electrospinning involves the simultaneous extrusion of two different solutions through a concentric nozzle, resulting in fibers with distinct core and shell regions. This technique is beneficial for incorporating hydrophobic bioactive compounds into CS-based fibers [82,83,84]. An extended shelf-life from 9 to 15 days at a nisin concentration of 8 mg/mL was reported [85].

Post-processing strategies like crosslinking are also essential for improving water resistance. Non-toxic crosslinkers such as genipin and citric acid are preferred for food contact. A CS/gelatin film crosslinked with genipin exhibited enhanced water resistance, mechanical strength, and UV-blocking properties [86]. However, excessive crosslinking—e.g., using oxidized sucrose—can reduce antimicrobial activity by binding amino groups vital for bacterial inhibition [87]. Thus, a careful balance is required to optimize both stability and bioactivity in CS-based packaging.

### 2.2. Polymer Blending Strategies for Electrospun CS Nanofibers

To enhance the electrospinnability of CS, one of the most effective strategies is polymer blending. CS is often combined with synthetic biodegradable polymers such as polyvinyl alcohol (PVA), polyethylene oxide (PEO), polycaprolactone (PCL), polylactic acid (PLA), and polyethylene glycol (PEG) [88,89], as summarized in Table 2. These polymers can modulate solution viscosity and enhance the electrospinning process.

PVA is one of the most commonly used copolymers with CS [90]. The hydrophilic nature of PVA and its compatibility with CS facilitate the formation of uniform nanofibers with improved mechanical properties [91]. Studies have demonstrated that CS/PVA nanofibers exhibit enhanced tensile strength (~5–8 MPa) and elongation at break between 13% 17% [92]. Similarly, the incorporation of PEO has been used to reduce the viscosity of the CS solution, enabling smoother electrospinning processes and yielding nanofibers with desirable morphological characteristics [93,94]. Additionally, PEO contributes to the flexibility and hydrophilicity of composite fibers [95]. When blended with CS, PCL enhances the electro-spinnability of the solution and improves the mechanical strength and flexibility of the resulting nanofibers. Studies have demonstrated that PCL/CS electrospun nanofibers exhibit a homogeneous fibrous microstructure with average diameters ranging from 250 to 270 nm [96]. The incorporation of CS into PCL matrices has been shown to reduce surface roughness by up to 240% compared to pure PCL films, imparting stable hydrophobic properties with water contact angles remaining steady at 107° after 20 s [97]. Additionally, these nanofiber films exhibit good biocompatibility, making them suitable for applications in food packaging where contact with consumables is a consideration.

**Table 2:** Synthetic biodegradable polymers blended with CS.

Synthetic Polymer	Molecular Weight (Mw)	Characteristics and Role in Blend	Notable Examples	Experimental Parameters	Refs.
Polyvinyl alcohol (PVA)	≈115 kDa (Mw; grade ~98–99% hydrolysis)	Forms hydrogen bonds with CS, improves spinnability, mechanical strength, and flexibility.	CS/PVA (90% CS) electrospun smooth, homogeneous fibers used as antimicrobial layers in meat packaging.	CS: DDA ≈ 91.5%, 0.05% (*w*/*v*); solvent: 2% acetic acid (*v*/*v*)	[98]
Polyethylene oxide (PEO)	≈1000 kDa (Mw)	Enhances electrospinnability by chain entanglement and surface tension reduction; food safe.	Inner CS–PEO nanofiber layer (90% CS) in active packaging reduced bacterial growth on raw meat.	CS: MW 102 kDa, DA 88%, 5% (*w*/*v*) in 0.5 M acetic acid	[61]
Polylactic acid (PLA)	≈40 kDa (Mw)	Improves tensile strength and lowers oxygen and water vapor permeability.	PLA/CS electrospun layers added to films for enhanced barrier and antimicrobial functions.	CS: MW 890 kDa, ~8% (*w*/*v*); solvent: 50% acetic acid	[99]
Polycaprolactone (PCL)	≈80 kDa (Mw)	Adds durability and moisture resistance; suitable for food-contact (not fully edible).	Starch/PCL/CS mats combined antimicrobial bioactivity and structural integrity for food safety.	CS: MW 120 kDa, DDA 85%; concentration 3% (*w*/*v*); solvent: formic acid: acetic acid = 70:30 (*v*/*v*)	[100]
Polyethylene glycol (PEG)	≈1.5 kDa (PEG 400)	Adds elasticity (low MW); assists fiber formation (high MW); modulates release kinetics; may increase fiber moisture sensitivity if used in large amounts.	CS/PEG fibers loaded with Eos for controlled release applications.	CS: DDA ≈ 91.5%; concentration 0.05% (*w*/*v*); solvent 2% (*v*/*v*) acetic acid	[98]

In parallel, Table 3 summarizes how blending CS with various natural polymers enhances the electrospinning process and tailors fiber performance for active packaging. Natural polymers such as gelatin, starch, sodium alginate, and zein have also been utilized in dual polymer systems with CS [101,102,103]. Starch, being abundant and cost-effective, contributes to the biodegradability and edibility of composite fibers. It has been extensively studied for its application in edible films and coatings due to its excellent film-forming capabilities. However, native starch films are brittle and sensitive to moisture [104,105]. To address these issues, modifications such as plasticization and blending with other biopolymers like CS have been employed [106]. CS-starch composite films exhibit enhanced mechanical properties, reduced water vapor permeability, and improved flexibility, making them suitable for food packaging applications [107]. Xu and coworkers prepared CS–corn starch films at a 2:1 starch: CS ratio and found these films achieved a tensile strength of 40.25 MPa (vs. ~25 MPa for pure CS), elongation at break over 61%, and water vapor permeability reduced from 52.7 to ~46.4 g·m^−2^·h^−1^. These enhancements highlight the synergetic benefits of blending CS with starch for creating mechanically robust, flexible, and moisture-resistant edible films.

Gelatin, derived from the partial hydrolysis of collagen, is a protein-based biopolymer known for its excellent film-forming ability, transparency, and biodegradability [113,114]. Gelatin-based films exhibit good oxygen barrier properties but are inherently hydrophilic, resulting in poor water vapor barrier performance [115,116]. Blending gelatin with CS can synergistically enhance the mechanical strength and water resistance of the resulting films [113,117]. For instance, gelatin–CS nanocomposite films were developed and reinforced with 1 wt% NiO nanoparticle, observing a rise in tensile strength from 12.32 ± 0.37 MPa (gelatin–CS control) to 26.61 ± 2.00 MPa. It is nearly a 2.2-fold increase alongside improved moisture barrier performance under water immersion, demonstrating significantly enhanced film robustness and hydration stability. Such composite films are edible and have been utilized in packaging applications to extend the shelf life of various food products such as strawberries, fresh-cut apples, and salmon filets. Sodium alginate, extracted from brown seaweed, is a hydrophilic polysaccharide known for its gel-forming ability and biocompatibility [118,119]. Recent studies have focused on optimizing sodium alginate-CS edible films incorporated with sea fennel by-product extract, aiming to enhance their antioxidant activity and suitability for food packaging applications [120,121].

Zein, a prolamin protein extracted from corn, is characterized by its hydrophobicity and excellent film-forming properties [122]. Zein-based films exhibit good barrier properties against moisture and oils, making them suitable for coating applications in the food industry. However, zein films are brittle and require plasticizers to improve their flexibility [123]. Pure zein films are brittle at room temperature, but the addition of plasticizers such as glycerol, PEG, and reducing sugars like fructose, galactose, and glucose at ratios up to 0.7 g plasticizer per g zein has been shown to significantly enhance film flexibility and reduce brittleness without compromising food safety. Consequently, combining zein with CS can result in composite films with balanced mechanical properties and enhanced functionality [124,125].

### 2.3. Inorganic Additive Strategies for Electrospun CS Nanofibers

Although CS films or fibers offer biocompatibility and antimicrobial properties, they often suffer from limitations such as low mechanical strength and inadequate barrier performance [126,127]. Beyond polymer blending, the incorporation of inorganic nanomaterials presents a powerful strategy to enhance the functionality of CS-based electrospun nanofibers for food packaging. Recent studies have focused on integrating food-safe inorganic additives, such as zinc oxide (ZnO), titanium dioxide (TiO_2_), nano-silica, and various clays, to form CS-based nanocomposites for active packaging [128,129,130,131,132].

Widely studied inorganic materials include metal oxides (e.g., ZnO, TiO_2_, CuO, CeO_2_), metal nanoparticles (e.g., Ag, Cu), and layered silicates such as montmorillonite (MMT) and halloysite nanotubes (HNTs) [133,134,135,136]. Among them, ZnO is especially favored due to its Generally Recognized as Safe (GRAS) status and its well-documented broad-spectrum antimicrobial efficacy [137]. It has been proven to significantly extend the shelf life of food products, such as prolonging the shelf life of meat and salmon by up to 14 days [138]. TiO_2_ nanoparticles contribute both potent UV-blocking capabilities and photocatalytic bactericidal effects. The inclusion of just 0.6 wt% TiO_2_ in a CS/PVA blend substantially increased tensile strength and Young’s modulus while simultaneously showing notable antimicrobial activity [139,140]. Specifically, tensile strength (TS) increased from 44.64 ± 1.49 MPa (neat CS) to 46.79 ± 1.65 MPa, while elongation at break (EAB) more than doubled, rising from 5.09 ± 0.38% to 12.26 ± 0.41%. Although Young’s modulus was not explicitly reported, the enhanced TS and EAB indicate a more ductile yet stronger film matrix. These composite films also exhibited strong antibacterial activity and successfully preserved cherry tomatoes during a 15-day storage period.

Other metal oxides, such as copper oxide (CuO) and cerium oxide (CeO_2_), have shown promise in enhancing antimicrobial efficacy, thermal stability, and mechanical integrity of CS nanofibers [141]. Magnesium oxide (MgO) and calcium carbonate (CaCO_3_) have also been explored for their UV-shielding and antibacterial potential, or as functional fillers in edible film formulations [142,143,144]. Additionally, bio-derived nanoparticles such as calcium phosphate (hydroxyapatite) from bone or shell sources offer inherently safe reinforcement for CS matrices, though their use in electrospun packaging remains limited [145,146].

Metal nanoparticles, particularly silver (Ag) and copper (Cu), have been extensively investigated for their potent antimicrobial activity when embedded in CS-based nanofibers [140,147,148]. Their biocidal effects are primarily attributed to the sustained release of Ag^+^ or Cu^2+^ ions and the generation of reactive oxygen species, which disrupt microbial membranes, proteins, and DNA [149,150]. For example, silver nanoparticles incorporated into CS matrices have demonstrated broad-spectrum antimicrobial efficacy against both Gram-positive and Gram-negative bacteria [151]. Similarly, copper and copper oxide nanoparticles have demonstrated strong antimicrobial properties [152].

However, despite their effectiveness, the use of silver and copper nanoparticles in food packaging is limited due to concerns regarding edibility, potential toxicity, and the risk of nanoparticle migration into food [153]. For example, AgNPs, a commonly used inorganic additive, may migrate from packaging materials into food either as ionic Ag, nanoparticles, or more complex species. Migration levels vary depending on the packaging matrix and food type. A study on AgNP migration from packaging into boneless chicken breasts reported levels ranging from 3 to 5 $\mu g/{\mathrm{dm}}^{2}$ [154]. Similar levels were observed in other commercial plastic containers, with migration values between 3.0 and 3.4 $\mu g/{\mathrm{dm}}^{2}$ [155]. In tests simulating real food contact conditions, total Ag migration from FresherLonger bags into 50% ethanol and 3% acetic acid simulants was measured at 0.2 and 0.4 $\mu g/{\mathrm{dm}}^{2}$, respectively [153]. Consequently, metal nanoparticles are typically restricted to non-edible packaging components or applied as coatings on outer layers to preserve their functional benefits while avoiding direct interaction with food products.

Clay minerals, particularly MMT and HNTs, have been extensively employed as functional fillers in CS-based electrospun nanofibers due to their natural abundance, biocompatibility, and favorable regulatory status [156,157]. Materials such as bentonite and calcium montmorillonite are recognized as GRAS and approved by the U.S. Food and Drug Administration (FDA) for use as carriers and anti-caking agents in food systems [158,159]. When incorporated into CS matrices, these layered silicates significantly enhance performance by introducing tortuous diffusion pathways that hinder the transmission of oxygen and water vapor, thereby improving the barrier properties of the resulting nanofibers [133]. Moreover, strong interfacial interactions, such as hydrogen bonding and electrostatic attraction, between the CS and silicate layers reinforce the fiber network, leading to significant improvements in mechanical properties. For instance, the addition of ~5 wt% MMT to CS/PVA nanofibers has been shown to substantially increase Young’s modulus, tensile strength, elongation at break, and overall toughness [39,160]. Electrospun CS/PVA nanofibers reinforced with 5 wt% MMT exhibited an increase in Young’s modulus from 190 MPa to 858 MPa and tensile strength from 5.26 MPa to 15.45 MPa following nano clay incorporation. Simultaneously, elongation at break and overall toughness were also markedly improved. Specifically, elongation increased from 0.65 MPa to 2.2 MPa at the same loading, reflecting considerable enhancement of fiber extensibility and resilience in the nanocomposite matrix.

HNTs also contribute unique functionality by acting as nanocarriers for active agents, such as antioxidants, antimicrobials, and EOs, enabling controlled and sustained release over time [161,162]. This loading capacity makes HNTs particularly attractive for developing active food packaging systems with extended functional lifespans. Wu et al. [163] reviewed halloysite–polysaccharide nanocomposites and reported that HNT lumen can accommodate substantial payloads: the maximum hydrogen storage capacity reached ~2.8 wt% in drug-loaded composites, while methane storage was measured at 50.6 mg/mL following lumen enlargement and modification. Such significant loading highlights the ability of HNTs to act as controlled-release reservoirs, and when combined with biopolymer coatings, allows release of active agents (e.g., antimicrobials, antioxidants) over prolonged periods, and is ideal for active packaging applications.

Additionally, the use of organically modified clays, such as lecithin-treated MMT, has been reported to improve dispersion within biopolymer matrices and facilitate uniform fiber morphology during electrospinning. Beyond mechanical and barrier enhancements, clay-based fillers also show potential in absorbing ethylene and volatile organic compounds, contributing to delayed ripening and shelf-life extension [164]. For example, alkali-treated halloysite nanotubes (HNTs) incorporated into packaging matrices exhibited strong ethylene removal performance: in static sorption tests, raw HNTs adsorbed 6.36 mL/g, outperforming palladium-based scavengers (4.16 mL/g) and commercial alternatives. When formulated into a 5 wt% HNT/CS-based nanocomposite film, ethylene adsorption reached ~0.60 mL/g at 1 bar, effectively prolonging cherry tomato and banana shelf life by approximately 7 days [165]. Collectively, the incorporation of clay minerals into CS nanofiber systems offers a multifaceted approach to enhancing mechanical integrity, moisture and gas resistance, and the delivery of bioactive agents, making them up-and-coming candidates for next-generation sustainable food packaging applications.

Silica (SiO_2_) has also garnered considerable attention as a functional additive in CS-based electrospun nanofibers, particularly in its amorphous nanoparticulate or colloidal sol forms [147]. Amorphous silica is commonly used as an anti-caking agent and carrier in food formulations [166]. When incorporated into CS matrices, silica nanoparticles enhance the mechanical strength and structural integrity of the fibers through physical reinforcement and interfacial adhesion [167,168]. It is also an effective nanocarrier for hydrophobic bioactive compounds such as EOs. For instance, Eos such as basil and oregano, which exhibit antimicrobial and antioxidant properties but suffer from poor aqueous solubility and volatility, can be encapsulated within SiO_2_ nanoparticles before being blended with CS [169]. This strategy enhances the dispersion stability of oils within the polymer matrix, facilitating controlled release and resulting in significantly improved functional efficacy compared to the use of free oils alone [170,171]. CS, silica, and oil composite systems have demonstrated prolonged antimicrobial action and higher antioxidant activity, which are highly desirable for active food packaging. Similar encapsulation and dispersion enhancements have also been reported in other biopolymer matrices incorporating silica, underscoring its versatility and broad compatibility [172,173]. The integration of inorganic fillers into CS electrospun nanofibers can be accomplished through several distinct approaches, each influencing the final nanostructure, filler dispersion, and functional performance of the resulting composite [80,174].

### 2.4. Incorporation Methods

Selection of an appropriate incorporation method is crucial for achieving desirable physicochemical and bioactive properties in food packaging applications. One of the most widely employed methods is solution blending, wherein pre-synthesized inorganic nanoparticles or colloidal dispersions are directly mixed into the CS spinning solution. This approach is frequently facilitated using co-spinning polymers, such as PVA, PEO, or cellulose acetate, which enhance electrospinnability and matrix compatibility [175,176,177]. For instance, synthesized ZnO nanoparticles using a sol–gel method and incorporated 0.5 wt% ZnO into a 1 wt% CS/10 wt% PVA blend before electrospinning. This approach resulted in uniform fiber morphology with well-dispersed ZnO nanoparticles, a reduction in average fiber diameter from approximately 230 nm to 180 nm, enhanced antibacterial activity against *E. coli*, and an increase in tensile strength from 3.1 MPa to 5.4 MPa [178]. Solution blending is advantageous due to its simplicity and scalability. However, it often suffers from limitations related to nanoparticle aggregation and limited control over filler positioning within the fiber matrix.

To overcome such limitations, in situ, sol–gel, and precipitation techniques have been explored to enable the direct formation of nanoparticles within the polymer matrix [179]. In these methods, inorganic precursors such as metal alkoxides or salts (e.g., titanium isopropoxide, zinc nitrate) are added to the spinning solution and undergo hydrolysis and condensation either during or after electrospinning [180]. This process yields finely dispersed inorganic phases with a reduced risk of agglomeration and enhanced polymer-filler interfacial interactions [181]. For example, Gupta et al. [182] performed in situ hydrolysis of titanium isopropoxide during the electrospinning of CS/PEO solution, leading to the formation of uniformly distributed TiO_2_ nanoparticles within the CS fibers. This modification enhanced thermal stability, as indicated by a ~12 °C increase in melting temperature (T_m_), and increased Young’s modulus by 40%, demonstrating both mechanical reinforcement and functional enhancement while preserving nanofiber integrity. This strategy enhances mechanical reinforcement and functional performance while maintaining nanofiber integrity. In addition to pre-spinning incorporation, post-spinning modification techniques offer a flexible platform for functionalizing electrospun CS mats after fiber formation. One standard method involves immersing the electrospun mats in metal salt solutions (e.g., AgNO_3_, CuSO_4_, Zn(NO_3_)_2_), followed by chemical reduction using agents such as sodium borohydride (NaBH_4_), which leads to the deposition of metal nanoparticles on the fiber surface [183,184]. This approach enables the precise localization of bioactive fillers on the surface without interfering with the spinning process.

Alternatively, physical vapor deposition or low-temperature plasma sputtering can be used to coat CS fibers with thin, uniform layers of inorganic materials [185]. These methods are particularly valuable when heat-sensitive polymers or actives are involved, as they operate under low thermal loads. Furthermore, dielectric-barrier plasma treatments have been reported to graft polar functional groups onto CS surfaces, thereby improving the adhesion and stability of subsequently applied inorganic coatings [186]. Surface functionalization techniques such as layer-by-layer assembly or chemical grafting enable the precise engineering of fiber surfaces to incorporate inorganic fillers like clays or silica [187]. For example, montmorillonite platelets can be pre-treated with amphiphilic agents (e.g., lecithin or quaternary ammonium compounds) to enhance their dispersibility and affinity for the CS surface, then deposited via electrostatic or hydrogen-bond-driven assembly to form layered barrier coatings [188,189]. Such surface engineering not only enhances gas and moisture barrier properties but also contributes to mechanical strengthening and surface antimicrobial activity.

Each incorporation method has distinct advantages and trade-offs. Physical blending is straightforward and suitable for large-scale production, but may result in heterogeneous filler distribution and particle aggregation. In contrast, in situ, sol–gel synthesis offers superior dispersion and filler–matrix integration, albeit with more complex processing requirements. Coaxial and emulsion electrospinning enable encapsulation and release control, while post-treatment and surface functionalization methods allow for the independent tuning of surface characteristics without altering the bulk fiber properties. In practice, hybrid strategies that combine in situ synthesis with post-deposition techniques are increasingly being adopted to optimize the performance of composite nanofibers. Specific food packaging functionalities include mechanical reinforcement, controlled release of active agents, and enhanced barrier properties.

In summary, the integration of inorganic fillers into CS-based electrospun nanofibers provides a highly adaptable platform for designing next-generation active packaging materials. Through strategic selection of filler type, concentration, and incorporation method, it is possible to achieve synergistic enhancements in antimicrobial activity, UV protection, gas and moisture barrier properties, mechanical resilience, and even targeted chemical functionalities. Importantly, many of these materials can be sourced from GRAS-listed or food-contact-safe substances, thereby enabling their alignment with regulatory requirements and ensuring consumer safety [132,137,190,191]. For example, zinc oxide is explicitly listed as GRAS under 21 CFR 182.8991 and is permitted in food-contact applications, including adhesives, coatings, and can liners. Under U.S. law, substances added to food must satisfy the standard of “reasonable certainty of no harm” (21 CFR § 170.3/170.30) or be approved via Food Contact Notifications (FCNs) or Threshold of Regulation (TOR) exemptions. In the European Union, Regulation (EC) No. 1935/2004 provides a harmonized framework mandating that food contact materials must not transfer harmful levels of constituents, must preserve food composition and taste, and must follow good manufacturing practice (EC) No. 2023/2006. Furthermore, active materials like those incorporating nanoparticles or EOs must also be labeled as under Regulation EC No 450/2009 and must include a declaration of compliance and traceability documentation. By selecting components that are already FDA-GRAS or listed under EU frameworks, and by adhering to FCN/TOR procedures, Regulation (EC) No. 1935/2004, and GMP standards, developers can ensure that multifunctional active packaging films meet both safety and regulatory requirements for food contact use.

These hybrid nanofiber systems hold substantial promises for improving the freshness, safety, and quality of perishable food products in real-world packaging scenarios. Consequently, a focused analysis of their mechanical performance, including strength, stiffness, flexibility, and toughness, is presented in Section 3, as these properties fundamentally determine the functional viability of CS-based electrospun nanofibers in packaging applications.

## 3. Functionalities of CS-Based Electrospun Nanofibers

### 3.1. Mechanical Properties and Characterization Techniques

The mechanical properties of CS-based electrospun nanofibers are crucial for their food packaging applications. CS packaging films must exhibit sufficient strength, flexibility, and durability to protect food products during handling and storage. Mechanical reinforcement is usually achieved through the addition of rigid inorganic nanofillers, which compensate for the inherent mechanical limitations of electrospun CS. Many studies have demonstrated that the incorporation of inorganic fillers significantly enhances the mechanical performance of CS-based nanofibers [31,192,193]. For instance, CS/PVA electrospun fibers embedded with montmorillonite exhibited substantial increases in Young’s modulus, tensile strength, elongation at break, and overall toughness compared to unfilled counterparts [194]. Similarly, the inclusion of 0.6 wt% TiO_2_ into protein/CS composite films resulted in marked improvements in tensile strength and stiffness [139]. These enhancements are attributed to the formation of strong interfacial interactions between the polymer matrix and rigid filler particles, which improve load transfer and reduce polymer chain mobility. Enhanced mechanical robustness ensures that the packaging material can withstand handling, bending, and tearing during storage and transport, thereby maintaining its protective function throughout its shelf life.

The primary techniques employed to assess these properties are tensile testing, atomic force microscopy (AFM), Raman spectroscopy, X-ray Diffraction (XRD), and Fourier-transform infrared (FTIR) spectroscopy. Each provides complementary insights into the mechanical behavior of the nanofibers.

Tensile testing is the primary method for assessing mechanical properties, including ultimate tensile strength (UTS), Young’s modulus (elastic modulus), elongation at break, and toughness. Standard uniaxial tensile tests are used on electrospun mats or films to quantify these metrics. Typically, pure CS nanofiber mats exhibit low tensile strength and limited elongation, which can restrict their use in packaging. In a study by Gu et al. [195], the mechanical properties of CS nanofiber mats were evaluated, revealing that as-spun mats exhibited a tensile strength of 2.41 MPa, an elastic modulus of 0.61 MPa, and an elongation at break of 14.1% [195]. Post-treatment processes such as neutralization and sonication were found to influence these properties. The neutralized mats showed increased tensile strength and elastic modulus but reduced elongation at break, indicating a trade-off between stiffness and flexibility. For instance, an as-spun CS/PVA nanofiber membrane exhibited a UTS in the range of ~3–6 MPa with an elongation below 10% [90]. Blending CS with a more ductile polymer, such as PVA, or adding reinforcing agents can significantly alter these values. Olvera Bernal et al. observed that increasing CS content (up to an optimal point) in a PVA/CS blend raised Young’s modulus, but too much CS reduced elasticity [90]. Specifically, Young’s modulus increased from approximately 300 MPa in lower-CS samples to around 648 MPa at the optimal composition (~3.5 wt% CS), after which further CS enrichment reduced elasticity. Correspondingly, tensile strength and elongation at break peaked at this composition before declining, highlighting the trade-off between stiffness and flexibility when varying CS content in PVA-based nanofibers. Conversely, other studies report that higher PVA fractions improve flexibility while CS addition can increase stiffness up to an optimal ratio [39,196]. Generally, an optimal CS-to-PVA ratio (often around 20:80 *w*/*w*) maximizes tensile strength and modulus without sacrificing elongation [197]. Table 4 summarizes representative tensile properties from recent studies:

AFM offers nanoscale insights into the surface morphology and mechanical properties of fibers. AFM imaging in tapping or non-contact mode can produce a 3D topography of CS nanofibers, revealing fiber diameter, surface texture, and roughness with nanometer resolution. Researchers have used AFM to measure the surface roughness (Ra) of individual fibers and entire mats. For instance, Langwald et al. [201] measured roughness along the CS fiber axis and found average Ra on the order of 0.5–8 nm for various electrospun fibers. Such low roughness at the single-fiber scale indicates relatively smooth fiber surfaces. However, at the mat level, the entangled fibers create a much larger apparent roughness (in the order of microns) when measured over a wider area. AFM height maps in this work show that the color-coded topography directly visualizes fibers as raised features on the substrate. Beyond imaging, AFM can perform nano-indentation or force mapping on electrospun fibers to assess local mechanical properties. In this study, AFM was operated in quantitative nanomechanical mapping (QNM) mode to measure local properties of CS nanofibers. Before data acquisition, the cantilever was calibrated to determine the precise spring constant and tip radius. The tip was then brought into contact with the nanofiber surface to perform force–distance spectroscopy at multiple pre-defined points. During each measurement cycle, the tip indented the surface with a controlled maximal force and then retracted, generating a force–displacement curve at each point. From these curves, mechanical parameters such as Young’s modulus, adhesion force, and deformation were calculated using established contact mechanics models (e.g., Hertz, Derjaguin–Muller–Toporov (DMT), or Johnson–Kendall–Roberts (JKR), depending on adhesion and material characteristics [202,203,204]). These point-wise measurements were then compiled into a nanomechanical map, enabling spatial resolution of local stiffness and surface interactions across the fiber surface. Although such measurements are challenging (due to fiber compliance and substrate effects), they have been reported for hydrated biopolymer fibers and hydrogels [205]. In general, AFM studies of CS nanofibers confirm that surface roughness and stiffness can be tuned by fiber composition:, e.g., coating or blending fibers with nanoparticles tends to increase surface roughness (due to protruding particles) and can also locally stiffen the fiber surface [206]. Surface roughness can influence hydrophobicity and cell interactions. A moderate increase in roughness has been correlated with lower water contact angles (indicating a more hydrophilic surface) in some CS/PVA mats, likely due to the nanoscale texture affecting wetting [207].

Raman spectroscopy is a powerful tool for probing the molecular structure and interactions in CS-based nanofibers. CS exhibits characteristic chemical bands (e.g., C–H, amide, and saccharide ring vibrations). When CS is blended or chemically modified, shifts in these bands can indicate the formation of interactions. For example, in electrospun CS/ polyvinylpyrrolidone (PVP) fibers, Raman analysis revealed that the position of the carbonyl (C=O) stretching band of PVP shifted when CS was added due to hydrogen bonding between CS’s –NH/–OH groups and PVP’s C=O [208]. A significant downshift of the C=O Raman band is indicative of strong intermolecular hydrogen bonding in polymer blends [209]. Grant et al. [210] demonstrated that both Raman and FTIR were sensitive to these interactions: they observed a downshift of the PVP carbonyl frequency and increased intensity of CS’s C–O stretching, confirming the formation of a CS–PVP hydrogen-bonded complex. FTIR analysis revealed a downshift of the PVP carbonyl stretch from ~1661 cm^−1^ (pure PVP) to ~1557 cm^−1^ in the CS/PVP blend. This shift toward lower wavenumber indicates that the C=O groups of PVP are engaged in hydrogen bonding, most likely donating electron density to CS’s –NH_2_ or –OH groups, reducing the bond strength of the carbonyl group. Raman spectra showed a linear increase in intensity of the CS C–O stretching band (around 1030–1076 cm^−1^) with increasing CS content. This suggests enhanced molecular interactions and confirms that CS is being incorporated within a hydrogen-bonded CS–PVP network. Raman microscopy can also map the distribution of components in composite fibers. This approach has been applied to confirm the uniform distribution of curcumin in gelatin/CS/curcumin fibers (curcumin exhibits distinctive Raman peaks) or to differentiate between polymer phases in core–shell fibers [211].

Moreover, Raman spectra can indicate the crystallinity of polymer phases: CS typically exhibits broad Raman peaks due to its semicrystalline nature. However, the presence of a crystalline additive (such as a drug or nanocrystal) may result in sharp peaks. Changes in peak width and position can thus signal crystalline changes or molecular orientation. In one study, the ratio of specific Raman peak intensities (I_(2930)_/I_(2980)_) was used as a quantitative measure of CS content in a PVP/CS fiber blend [210,212]. Overall, Raman spectroscopy in the 300–1800 cm^−1^ range (fingerprint region) and higher wavenumber region can identify functional groups, assess crystallinity, and detect specific interactions or the presence of additives in CS nanofibers [213]. The downshifts often signal hydrogen bonding or complex formation, sharper peaks indicate increased crystalline or molecular order, and intensity variations can quantify the degree of polymer-additive interaction. In higher-wavenumber spectra (above 2800 cm^−1^), CH and NH stretching bands yield insights into backbone polarity and hydrogen-bonding dynamics. Altogether, Raman provides a unique “molecular fingerprint” that simultaneously confirms composition, assesses structural order, and identifies specific chemical interactions vital for optimizing active packaging nanofibers. This information complements FTIR and XRD by providing a molecular fingerprint that is sensitive to both chemical composition and molecular order.

XRD is used to analyze the crystalline versus amorphous phases in electrospun nanofibers. Pure CS is mostly amorphous (especially in electrospun form or when neutralized), typically showing a broad diffuse diffraction peak around 2θ ≈ 20° attributable to its semicrystalline domains [214]. Blending CS with other polymers or fillers often results in changes to the diffraction pattern. For instance, electrospun PCL/CS hybrid fibers illustrate this: neat PCL is a semi-crystalline polymer and exhibits sharp XRD peaks at 21.5° and 23.6° 2θ (from its (110) and (200) crystalline planes) [215,216,217]. In a PCL/CS blended nanofiber, XRD revealed that the intensity of PCL’s crystalline peaks was reduced and broadened compared to pure PCL, indicating a lower overall crystallinity due to the presence of CS [218]. Essentially, the CS disrupts PCL’s crystalline domain formation, yielding a more amorphous composite fiber structure. Kao et al. [219] reported that blending 50% CS into PCL fibers interfered with PCL’s crystal packing, consistent with a decrease in peak intensity on XRD. This kind of result is typical: the introduction of a second component (especially a non-crystalline one) often reduces the crystallinity of the electrospun matrix. Crystallinity is a major determinant of key material properties such as stiffness, strength, thermal resistance, and barrier performance. When crystallinity is reduced, the result is generally more flexible and ductile, but also less mechanically stiff and thermally stable, and more permeable to gases and moisture, because the amorphous regions allow easier diffusion. This shift represents a deliberate and often advantageous trade-off in active packaging applications, where enhanced flexibility and functional responsiveness can outweigh the loss in rigidity, yet the changes in strength, thermal tolerance, and barrier efficiency must be carefully balanced.

XRD is also valuable for detecting new crystalline phases (for example, salt crystals if CS is not fully de-acidified or crystalline nanoparticles in the fibers). If inorganic nanoparticles (ZnO, TiO_2_, clay, etc.) are incorporated, their distinct diffraction peaks can confirm successful embedding in the fibers [220]. In CS/ZnO nanofiber mats, one would see the ZnO’s wurtzite peaks superimposed on CS’s halo [221]. The absence or broadening of expected peaks can also indicate successful nanoscale dispersion (e.g., no large ZnO crystals formed, or the drug is molecularly dispersed rather than crystalline). Furthermore, XRD can monitor structural changes upon crosslinking. Crosslinkers like genipin or glutaraldehyde may induce or reduce crystallinity: for example, one study found that after crosslinking gelatin–CS fibers with genipin, the characteristic CS peak at ~20° became slightly less intense, suggesting the crosslinked network-constrained polymer chain arrangement was more amorphous [222]. This structural transition implies that the tightly packed CS chains, which confer rigidity and barrier properties, have been locked into more flexible, randomly arranged configurations by the genipin-induced crosslinked network.

FTIR spectroscopy is routinely employed to identify functional groups in CS nanofibers and to probe bonding interactions between CS and other components. When CS is blended or chemically modified, these FTIR spectral peaks can shift or change in intensity. A downshift (red shift) typically indicates new hydrogen bonding, which weakens bond strength and lowers vibrational frequency, such as CS’s –OH, –NH, or C=O when engaged in hydrogen bonding with additives or crosslinkers. Conversely, an intensity increase or peak broadening, especially in hydrogen-bonding regions, often reflects a higher proportion of bonded functional groups and more disordered amorphous character due to disturbed polymer packing. These spectral changes thus imply both chemical complexation and microstructural rearrangement, enabling researchers to confirm additive integration and quantify interaction strength. It is crucial for tailoring functionality in CS-based materials. Hydrogen bonding or electrostatic interactions often cause notable shifts: in CS/PVA or CS/PVP blends, researchers have observed that the carbonyl stretching band of the partner polymer shifts to a lower wavenumber in the FTIR spectrum, indicating H-bond formation with CS’s –OH or –NH groups [223]. Grant et al. [210] reported a downshift of the PVP carbonyl band (from ~1660 ${\mathrm{cm}}^{-1}$ to lower frequency) upon mixing with CS, alongside an increase in the C–O stretch region of CS—both evidence of intermolecular hydrogen bonds. The degree of shift can even be correlated with bond strength: carbonyl bands below ~1660 ${\mathrm{cm}}^{-1}$ suggest strong H-bonding in polymer complexes. FTIR is also crucial for confirming chemical reactions such as crosslinking or functionalization. For example, if CS nanofibers are crosslinked with glutaraldehyde, FTIR will show the appearance of imine bonds (C=N stretching around 1640–1650 ${\mathrm{cm}}^{-1}$) formed by Schiff base reaction between CS’s amine and glutaraldehyde carbonyl [224]. Similarly, new peaks can confirm the presence of additives; for example, the incorporation of polyphenols or EOs in CS nanofibers can be validated by their characteristic aromatic peaks (e.g., peaks around 1510 and 1600 ${\mathrm{cm}}^{-1}$ for aromatic ring vibrations of plant polyphenols) [54]. In one study, CS nanofibers loaded with 5-fluorouracil (an anticancer drug) showed additional peaks in FTIR corresponding to the drug, and intensity changes in CS’s amide bands indicated interactions between the drug and polymer [225].

Furthermore, FTIR can semi-quantitatively assess composition. By monitoring the intensity ratios of specific bands (for example, the amide II band of CS versus a reference band of a second polymer), one can confirm successful blending or even estimate the blend ratio in the fibers. An example from a composite fiber study: even 1% CS in a PVP fiber caused new FTIR bands to appear in the 1000–1100 cm^−1^ range (C–O–C and C–C stretches), and increasing CS content led to linear increases in those bands’ intensity [210]. This demonstrates FTIR’s sensitivity to small amounts of CS. Additionally, FTIR is often used to check the degree of acetylation of CS by the ratio of amide I to polysaccharide peaks and to ensure the fiber has been neutralized (the disappearance of the −COO ^−1^ band of CS acetate around ~1550 cm^−1^ would indicate removal of acetic acid) [226]. Overall, FTIR provides a molecular “fingerprint” confirming that CS functional groups are present and engaged in new interactions in the electrospun fibers.

### 3.2. Thermal Stability and Barrier Properties

The thermal stability of CS-based electrospun nanofibers is crucial for food packaging applications that involve exposure to elevated temperatures. It has been demonstrated that blending CS with other polymers can enhance its thermal properties [227]. For instance, incorporating PVA and tannic acid (TA) into CS nanofibers, followed by crosslinking with glutaraldehyde (GA), significantly improved thermal stability [196]. The decomposition temperature increased from 225 °C to 310 °C, indicating enhanced resistance to thermal degradation [228]. Similarly, the addition of eucalyptus EO to gelatin-CS nanofibers resulted in improved thermal stability, as indicated by thermogravimetric analysis [229]. Specifically, the onset degradation temperature shifted from approximately 280 °C to 295 °C, and the maximum degradation rate temperature (T_max_) increased by about 5–10 °C in EO-containing fibers. These shifts indicate enhanced polymer matrix stability, attributable to the oil’s ability to interact with the biopolymer network, delaying thermal decomposition and suggesting improved resilience under heat stress—an important trait for food-packaging applications where thermal resistance may be required.

Moisture resistance is another critical factor for food packaging materials. CS-based nanofibers inherently possess hydrophilic characteristics, which can be modified to enhance moisture barrier properties. Crosslinking CS with agents like glutaraldehyde GA has been shown to reduce water vapor permeability and improve water resistance. For example, Gierszewska et al. [230] reported that CS–MMT composite films crosslinked with 0.99 mg GA per 150 mL solution, in the presence of 10 wt% glycerol, exhibited a WVP of 3.60 g·mm/kPa·day·m^2^, compared to significantly higher values in non-crosslinked controls. This demonstrates that GA crosslinking effectively decreases hydrophilicity and strengthens film integrity. It is a key advantage for food packaging applications. In the case of PVA/CS/TA nanofibrous membranes, crosslinking resulted in a significant decrease in water vapor permeability, thereby enhancing the material’s suitability for food packaging applications [228]. Additionally, the incorporation of high-molecular-weight CS into cellulose nanofiber films decreased water absorption and solubility, further enhancing moisture resistance. These enhancements in thermal and moisture resistance, achieved through polymer blending and crosslinking strategies, underscore the potential of CS-based electrospun nanofibers in developing practical and sustainable food packaging solutions.

The antioxidant activity of CS can be significantly enhanced by incorporating various bioactive compounds into electrospun nanofibers. For instance, the incorporation of curcumin, a polyphenolic compound known for its potent antioxidant activity, into gelatin/CS nanofibers has been shown to enhance the antioxidant properties of the resulting nanofibers [211]. The presence of curcumin facilitated stronger hydrogen bonding within the polymer matrix, thereby increasing the antioxidant capacity of the nanofibers. DPPH radical scavenging activity increased from 8.95 ± 0.64% in the control (curcumin-free) fibers to 25.53 ± 0.52%, 41.32 ± 0.60%, and 51.16 ± 0.59% with curcumin loadings of 0.1%, 0.2%, and 0.3% (*w*/*w*), respectively. This enhancement was evidenced by a significant increase in DPPH radical scavenging activity, indicating the potential of these nanofibers in active food packaging applications [211]. Similarly, the incorporation of EOs, such as eucalyptus EO, into gelatin-CS nanofibers has been shown to enhance antioxidant activity. The EOs contribute to the antioxidant properties by providing additional phenolic compounds that can scavenge free radicals [231]. This synergistic effect between CS and Eos results in nanofibers with enhanced antioxidant capacity, suitable for extending the shelf life of food products.

Barrier properties have been substantially improved by the incorporation of inorganic materials in another area. High-aspect-ratio fillers, such as montmorillonite platelets and halloysite nanotubes, create tortuous diffusion pathways within the polymer matrix, thereby significantly reducing permeability to oxygen and water vapor. These enhancements are essential for extending food shelf life by reducing moisture loss, oxidative spoilage, and microbial contamination. For instance, the incorporation of 2–6 wt% halloysite nanotubes loaded with chlorogenic acid into CS/PCL nanofibers has been shown to markedly reduce water vapor transmission rates [232,233]. In addition, strong electrostatic interactions and hydrogen bonding between the CS matrix and clay surfaces can further compact the fiber structure, decrease porosity, and enhance gas barrier performance [234].

### 3.3. Antimicrobial and Antiviral Properties

The antimicrobial efficacy of CS is influenced by factors such as its MW, DD, and environment. For instance, moderate MW CS (≈14,000–94,000 Da) exhibits optimal bactericidal activity, as polymers within this range balance wall penetration and surface interaction; in contrast, very high MW CS tends to have reduced efficacy due to limited diffusion through bacterial membranes. Furthermore, higher DD reflecting a greater proportion of free amino groups, correlates with stronger positive charge density, enhancing electrostatic interactions with negatively charged microbial cell surfaces and thereby increasing antimicrobial potency. Electrospinning CS into nanofibers enhances its antimicrobial activity due to the increased surface area and porosity, facilitating better interaction with microbial cells. The study [235] has demonstrated that CS-based electrospun nanofibers exhibit significant antibacterial activity against both Gram-positive and Gram-negative bacteria. For instance, electrospun CS/polyethylene oxide (PEO) nanofibers have shown effective inhibition of *Escherichia coli* and *Staphylococcus aureus*, common foodborne pathogens [93]. Quantitative assays revealed a 1.2-log reduction in *Escherichia coli* and a 1.5-log reduction in *Staphylococcus aureus* after 24 h exposure to the fibers, substantially higher than untreated controls. The incorporation of bacterial cellulose into CS/PEO nanofibers further enhances their mechanical properties and antimicrobial efficacy, making them suitable for active food packaging applications.

Additionally, the integration of bioactive compounds into CS-based nanofibers has been explored to augment their antimicrobial properties. For example, the incorporation of jaboticaba peel extract, rich in phenolic compounds, into CS/zein electrospun nanofibers resulted in bilayer films with enhanced antimicrobial activity against E. coli and *S. aureus* [93]. Specifically, the JPE-loaded bilayer exhibited inhibition halos of 8.77 ± 0.31 mm for *E. coli* and 9.32 ± 0.21 mm for *S. aureus*, while control films without extract showed no inhibition, clearly demonstrating the extract’s antimicrobial efficacy within the nanofiber matrix. These films not only exhibited improved barrier properties but also demonstrated potential as sustainable food packaging materials.

Beyond antibacterial activity, CS and its derivatives have shown promise in antiviral applications. The antiviral mechanism of CS is attributed to its ability to bind to viral particles, thereby inhibiting their attachment and entry into host cells. Moreover, CS can stimulate the host’s immune response, enhancing antiviral defenses. A recent study [236] has explored the antiviral potential of CS-based nanofibers in various applications. For instance, CS nanoparticles encapsulating antiviral agents, such as sofosbuvir, have demonstrated enhanced antiviral activity against hepatitis C virus genotype 4 (HCV-4) in vitro. Sofosbuvir was successfully encapsulated into CS nanoparticles (SCNPs; average size ~137 ± 34 nm, zeta potential +29 ± 9.6 mV, 80% encapsulation efficiency) and tested against hepatitis C virus genotype 4 (HCV-4) in Huh7 hepatoma cells. These nanoparticles not only improved the drug’s bioavailability but also reduced its cytotoxicity, highlighting the potential of CS-based nanocarriers in antiviral therapy.

In addition to antimicrobial protection, UV shielding is another critical functionality imparted by inorganic fillers. Both TiO_2_ and ZnO are well known for their high UV absorption capabilities. When incorporated into CS nanofiber mats, these oxides provide practical barriers against UV radiation, preventing the photodegradation of sensitive food components and packaging materials. This UV-blocking capacity helps maintain the color, nutritional quality, and oxidative stability of packaged products by reducing the photolytic breakdown of lipids and antioxidants. For example, CS–TiO_2_ composite nanofibers have demonstrated strong UV-protective effects, significantly reducing exposure-induced quality loss in fresh produce [237]. ZnO also contributes to UV filtering, although to a slightly lesser extent [137].

CS-based electrospun nanofibers exhibit a rich suite of functional properties that range from enhanced mechanical strength and tunable barrier performance to pronounced antimicrobial, antioxidant, and even antiviral activities. It is achieved through a lot of polymers, fillers, and active agents. These results underscore the versatility of nanofiber platforms in addressing diverse packaging requirements. The next section details how their tailored properties translate into practical uses in food packaging.

## 4. Applications of CS-Based Electrospun Nanofibers

**Figure 2:**
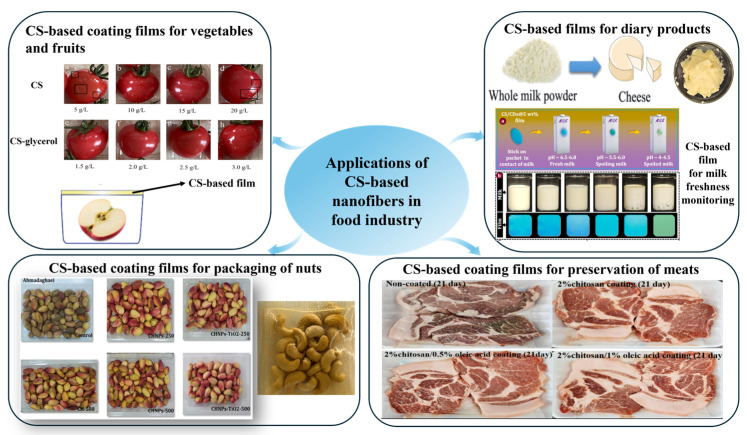
Applications of CS-based nanofibers in the food industry. Reprinted with permission from refs. [240,241,242,243,244,245,246,247]. Copyright Elsevier, 2025; Elsevier, 2024; Elsevier, 2024; Elsevier, 2024; MDPI, 2022; Elsevier, 2025; Elsevier, 2025; MDPI, 2025.

The applications of CS-based nanofibers in the food field mainly include food additives and food packaging materials. For food additives, CS or CS-based nanofibers are directly incorporated into foods such as vegetables, fruits, meat, dairy products, and nuts to improve their shelf life. For food packaging materials, there are two types of packaging: active packaging and intelligent packaging. Active packaging refers to protecting food against an external environment, such as antimicrobial films [238]. Innovative packaging enables food packaging materials to monitor the food and its environmental conditions [239].Figure 2 illustrates the diverse applications of CS-based nanofibers in the food industry, including preservation of fruits and vegetables, enhancement of dairy products, extension of meat shelf life and protection of nuts through antimicrobial and antioxidant coating.

### 4.1. CS-Based Nanofibers for Vegetables, Fruits, Salads, Fresh-Cut Foods

Vegetables, fruit, salads, and fresh-cut foods spoilage and post-harvest loss represent significant global challenges, resulting in diminished quality, nutritional value, and marketability of produce [248]. This kind of loss is attributed to multiple factors, including mechanical injuries, physiological disorders, microbial contamination, and unfavorable environmental conditions [249]. The conventional techniques, including refrigeration, cold storage, and modified atmosphere packaging (MAP), to mitigate those losses present some challenges, such as high cost and environmental problems [243]. Consequently, researchers are interested in alternative and more sustainable preservation methods for vegetables, fruit, salads, and fresh-cut foods. Edible coatings, especially those made from CS, have attracted researchers’ attention since this preservation method can extend the shelf life of vegetables and fruits during storage [250]. Currently, researchers have found that a CS-based coating on fruit and vegetables is effective in reducing a variety of harmful microorganisms, such as bacteria, fungi, and yeasts, and extending the shelf life of these products. Mahin et al. [251] assess the characteristics of CS obtained from shrimp shells and use it as the coating to prolong the shelf life of vegetables and fruits. They find that the concentration of CS results in the most significant shelf-life extension, with carrots, green tomatoes, tomatoes, cucumbers, and beans showing increases of 12, 21, 21, and 14 days, respectively.

Researchers have actively explored the incorporation of natural polymers, inorganic nanomaterials, and EOs into CS to develop CS-based nanofibers aimed at extending the shelf life of fruits and vegetables. For instance, Luo et al. [246] utilized taxfolin (Tax) and copper to develop CuTax nanozymes with strong free radical scavenging activity. These nanozymes demonstrated potent antibacterial effects by reducing bacterial viability and disrupting cell structures. When incorporated into CS to form composite coating films, CuTax significantly enhanced food preservation. Experiments using apples and bananas showed that the CuTax/CS coatings delayed spoilage by reducing weight loss, maintaining firmness, and lowering browning indices in bananas, supporting their potential for active food packaging applications. Zhang et al. [252] synthesized silver nanoparticles (AgNPs) using EMPO-oxidized nanocellulose (TCNF) as a green reductant and stabilizer, and subsequently integrated the TCNF-immobilized AgNPs into CS to fabricate antibacterial composite films. These CS/TCNF/AgNP films exhibited strong antimicrobial activity against *E. coli* and *S. aureus*, effectively reducing microbial contamination and preserving produce quality. In practical tests, the films extended the freshness of cherry tomatoes and strawberries by up to 12 and 6 days, respectively, highlighting their promise as active packaging materials for prolonging fruit and vegetable shelf life.

Besides vegetables and fruits, fresh-cut foods can also be preserved well by retaining their firmness and weight, as well as antimicrobial activity, modified gas permeability, and an enhanced physical barrier. For the retention of firmness and weight loss in fresh-cut foods, Khaliq et al. [253] found that water transmission and the transpiration process cause weight loss in fresh-cut foods, and consider edible coatings to hinder these processes. According to Jongsri et al. [254], CS-based coating can cover the stomatal apertures of fresh-cut fruits by slowing down the transpiration process and the respiration rate. Specifically, the experiment shows ‘Nam Dok Mai’ mango with CS-based coating film has only 11.61% weight loss, while the mango without a coating exhibits 13.61% weight loss in the 12 storage days. Therefore, CS-based preservation films can reduce the weight loss of fresh-cut fruits.

For antimicrobial activity, CS has been shown [255] to exhibit antimicrobial properties against various microorganisms like *Staphylococcus aureus* (*S. aureus*) and *Escherichia coli* (*E. coli*). The potential mechanism of antimicrobial properties is attributed to its chelating ability, whereby it selectively binds trace metal elements, leading to the inhibition of toxin production and microbial growth [256]. For modified gas permeability, one of the key mechanisms by which CS-based nanofiber coating can preserve fresh-cut fruit is by modifying gas permeability, which represents the ability for oxygen and carbon dioxide to pass through the membrane. For enhanced physical barriers, CS can form a physical barrier on the surface of fresh-cut foods, minimizing moisture loss and preventing dehydration. By limiting microbial penetration, the CS-based nanofiber films effectively reduce the risk of contamination and contribute to shelf-life extension of more than 13 days [257]. Fresh-cut salads easily generate foodborne bacteria. Therefore, rapid detection of foodborne bacteria in fresh-cut salads is essential for preventing foodborne bacterial outbreaks. Sang et al. [258] developed two types of magnetic nanoparticles (MNPs), including CS-coated MNPs and arginine-modified CS-coated MNPs, to capture multiple bacteria in the fresh-cut salad. They find that arginine-modified CS-coated MNPs function as a tool for rapid detection of foodborne bacteria via real-time PCR analysis following bacterial concentration, providing a promising approach to food safety applications.

In summary, CS-based nanofiber coating provides preservation for vegetables, fruits, salads, and fresh-cut foods by maintaining firmness and weight retention, exhibiting antimicrobial activity, modifying gas permeability, and enhancing the physical barrier.

### 4.2. Dairy Products

CS-based nanofibers have been used for contaminant detection in dairy products and packaging for the milk industry. For contaminant detection in milk, precise detection of contaminants such as bisphenol A, melamine, bacteria, drugs, antibiotics, toxins, heavy metals, and allergens is essential for food security. CS-based biosensors have been widely employed to detect contaminants in dairy products. Because melamine- and urea-contaminated cow’s milk poses serious health issues, including kidney stones and ulcers. Nasiri et al. [259] utilized the CS/graphene oxide (GO) nanocomposites to detect melamine. By comparing the detection results from the GO/CS nanocomposite to those from the standard HPLC method, they conclude that GO/CS sensors offer more desirable features in detecting melamine with high sensitivity 239.1 ${\mu M}^{-1}$, a linear range of 0.01–200 μM, an affinity constant of $1.73\times 10^{4}$ and an impressive limit of detection of 10 nM, making them a promising tool for accurately detecting melamine in various dairy products such as fluid milk, cheese, and yogurt. Feng et al. [260] also developed and utilized GO/CS as an electrochemical sensor for melamine detection. Their experimental work demonstrates excellent sensing of melamine with an ultrahigh sensitivity of 54.6 μA·μM^−1^·cm^−2^. In Ezhilan et al.’s study [261], the developed Pt/ZnO/AChE/CS bioelectrode is utilized to detect melamine and urea in cow’s milk. They validated that the proposed biosensor exhibits good recovery (99.96–102.22%), providing a tool for analysis of melamine and urea in cow milk samples. Besides melamine, bacteria such as Listeria, monocytogenes, and Salmonella are common contaminants in dairy products. Yue et al. [262] primarily focus on a set of nuclear magnetic resonance (NMR) biosensors based on an O-carboxymethyl CS target for gadolinium (Gd) probe, enabling the rapid detection of Salmonella in milk. The Salmonella in milk can be captured via the targeted probe through antigen–antibody interaction.

The other primary application of CS-based nanofibers in the milk industry is packaging material. Zhao et al. [263] incorporated the blood orange anthocyanins (BOA) and thyme oil (TO) emulsion into a CS-gum Arabic film matrix to develop multifunctional food packaging. The multifunction includes the basic properties, pH/volatile acid sensitivity, and functional characteristics. Finally, the developed multifunctional food packaging films effectively extend the shelf life of milk at room temperature, 25 °C, to 48 h, providing a new perspective for developing multifunctional food packaging films. Messinis et al. [264] utilized expired dairy products, such as milk and yogurt, to combine CS, glycerol, and squid ink to develop sustainable packaging material. They find that the edible CS-casein hydrogel packaging membranes exhibited promising mechanical properties and improved barrier properties compared to pure CS packaging materials. Specifically, the combinations of CS, glycerol, and squid ink cause a decrease in strength by 85.1% with significant enhancement of the strain by 467.5% in comparison to pure CS. For the improved barrier properties, their result shows that the CS-casein hydrogen films have zero oxygen permeability.

Inspired by such intelligent films, researchers have found that the ability to monitor product quality is an essential feature for packaging materials intended for dairy products. Chen and colleagues [265] compound the blueberry anthocyanin-derived cyanidin (BAC) with quaternary CS (QC) and gelatin (G) to form responsive food freshness packaging films (QC-G-BAC films), as the BAC solutions exhibit a noticeable color change under different pH conditions. Therefore, the addition of 5–15 wt% BAC to QC-G-BAC films could be employed for monitoring the freshness of pasteurized milk (from red to dark, earthy yellow). From this work, they conclude that the developed QC-G-BAC films offer a straightforward freshness indicator for dairy-related products. The idea of pH as an indicator of food freshness is widely used in intelligent packaging materials [266,267]. Overall, these findings demonstrate that CS-based nanofibers serve as pH-tracking materials, monitoring the real-time quality of dairy products [22]. CS-based indicator fibers or films can be compounded with other polymers to improve their mechanical properties, flexibility, stability, and sensing functionality. For example, Nguyen et al. [268] have developed composite intelligent films for freshness monitoring by blending CS with PVA and anthocyanins (ACNs). The resulting films demonstrated improved mechanical properties and uniformity compared to pure CS films, while still maintaining reliable colorimetric responses to fish spoilage. Such blending approaches enhance both the usability and effectiveness of CS-based freshness indicators, making them more suitable for practical food packaging applications.

In summary, CS-based nanofibers serve as practical tools for detecting contaminants in dairy products and freshness-responsive intelligent packaging materials for dairy products.

### 4.3. Meat and Seafood

CS exhibits antimicrobial properties and is effective against various microorganisms, including foodborne pathogens and fungi [269]. Based on their properties, CS and CS-based nanofibers are widely applied in the field of fresh meat and seafood products. That is because meat and seafood products are considered highly perishable and thus require an appropriate method to prevent spoilage during storage [270].

One application in the field of meat and seafood products is as a natural food additive for food preservation. Due to the non-toxic, antimicrobial, and antioxidant properties of CS, it is directly incorporated into various meat products as a food additive, effectively reducing microbial and pathogen growth in these products. For example, Lotfy et al. [271] directly mix CS with EOs of Mentha Piperita, Punica Granatum, Thymus Vulgaris, and Citrus Limon in olive oil to prepare the nanoemulsions. In all tested EOs, C. limon is the most active product with MIC of 1000 and 1500 mg/L against *E. coli* and *S. typhimurium*, respectively. The results show that Citrus Limon and its nanoemulsions have the best antibacterial performance. This work suggests that EO-CS nanoemulsions help extend the shelf life of meat products.

Additionally, Juneja et al. [272] mix the CS with thawed beef or turkey with distinct concentrations to investigate the CS’s effect on inhibiting the Clostridium Perfringens spore during abusive chilling of cooked ground beef and turkey. Their research results show that incorporating 3% CS into ground beef or turkey can reduce the potential risk of C. perfringens spore germination and outgrowth during abusive cooling from 54.4 to 7.2 °C in 12, 15, or 18 h. Soultos et al. [273] investigated the effect of CS and CS with nitrites on the microbiology of fresh pork sausage. The experimental work shows that the rate of lipid oxidation in fresh pork sausages is significantly decreased (the probability < 0.05) by the addition of increasing levels of CS, while samples containing both CS and nitrites show the lowest malondialdehyde (MDA) values. So, they conclude that CS can effectively reduce microbial growth, while the nitrites do not have a significant effect on inhibiting microbial growth in fresh pork sausage. There are other related studies where researchers add CS as a food additive to pork, chicken, and beef to achieve microbial growth reduction and extend the meat’s shelf life. In this section, we summarize the research studies on the application of CS as a food additive in various types of meat products, emphasizing its antimicrobial properties and its ability to suppress the growth of microorganisms and foodborne pathogens.

Apart from its direct incorporation of CS into meats, the other primary application in the meat and seafood products field is the development of CS-based nanofiber coating films for meat packaging and preservation. Esmaeili et al. [274] compared the effect of coating and nano-coating CS Lepidium sativum seed gum (CSG) on the shelf-life of beef during 18 days at cold temperatures. Their research results show that the composites with 66% CS and 33% CSG exhibit the best antioxidant and antibacterial properties. In addition, the antioxidant and antibacterial activities of nano-coating are generally higher than those of coating. Based on their research, the study suggests that a 66% CS:33% CSG blend is a safe and effective preservative for the active packaging of beef during refrigeration and freezer storage. Xiong et al. [275] developed a CS-gelatine edible coating system incorporating grape seed extract and/or nisin. They investigated the effect of the developed CS-based coating system on the preservation of fresh pork during cold storage. The thiobarbituric acid reactive substances value is used to evaluate the lipid oxidation, in which a higher value indicates a higher degree of lipid oxidation in the experiment. They conclude that the combination of 1% CS, 3% gelatine (CS-GEL), and 0.5% grape seed extract (CS-GEL-GSE) provides the most effective enhancement of antioxidant activities against meat oxidation. In addition, they find that the addition of nisin to CS-GEL-GSE does not further improve overall antimicrobial and antioxidant properties. Zheng et al. [276] examined the effects of CS coating compounded with oregano EO (OEO) on the microbial and physicochemical characteristics of chicken breast filets during cold storage. They find that CS+OEO coating not only inhibits the compounds causing unpleasant odors but also reduces the sensory properties of chicken breast, including appearance, color, and texture. Other recent papers, including CS mixed with rosemary EO (REO) [277], oregano EO and olive oil [278], and oregano oil and thyme oil [279], have shown that these systems can extend the shelf-life of rabbit meats, mutton, and beef, respectively. There are two general approaches to EO loading: passive incorporation and active, stimuli-responsive encapsulation. Passive systems often suffer from uncontrolled volatility and premature loss of antibacterial efficacy. In contrast, active systems utilize external triggers—such as changes in pH, temperature, or humidity—to modulate EO release. For instance, CS-based films and nanoparticles have been engineered to release EOs more rapidly under acidic conditions associated with food spoilage, enabling pH-triggered antibacterial action [280]. Temperature shifts during storage and distribution can also serve as a stimulus, allowing for thermally triggered release [281]. Moreover, the presence of specific enzymes produced by degrading food or microbes can be used to initiate EO diffusion, allowing microbial activity to trigger antimicrobial release precisely when needed [282]. These stimuli-responsive systems allow for more effective and sustainable use of EOs in food preservation by minimizing premature loss and enabling targeted delivery of antimicrobial agents. Apart from those EOs, CS is also incorporated with phenolic compounds, such as gallic acid and oleic acid, to extend the shelf life of fresh meats. Zheng et al. [283] incorporated gallic acid into CS to enhance the antibacterial and antioxidant properties of CS. Hoa et al. [244] blended CS with oleic acid to form a CS/oleic acid edible coating for extending the shelf life of fresh pork under aerobic packaging conditions. They find that the aerobic bacteria and *Pseudomonas* spp. counts, and total volatile basic nitrogen (TVBN) are almost two or three times lower in the CS/oleic acid-coated samples in comparison to samples without coating. This work suggests a potential application of CS/Oleic acid edible coating in the preservation of fresh pork.

### 4.4. Nuts

Nuts are also an important part of people’s daily lives. To address the quality deterioration of nuts, edible CS-based coating films could create a barrier to oxygen, carbon dioxide, and water, thereby minimizing microbial contamination of nuts [284]. Halloub et al. [242] develop self-healing food packaging materials for the preservation of cashew nuts, addressing the issues of damaged packaging materials. That is because the packing materials often deteriorate when transported, handled, and stored for an extended period. They combine the UV-triggered self-healing film containing omega-3 microcapsules with a CS–hemicellulose blend to form the coating films and investigate their mechanical properties, structural, thermal, and self-healing abilities. The developed omega-3 microcapsule/CS coating films, used as packaging materials, help stabilize the PH values of cashews during long-term storage. The non-coated samples demonstrate a rapid decline in PH value, while the coated cashew is 6.58, which is maintained for several days. Therefore, such coating films are applied to preserve cashew nuts, effectively extending their shelf life to 60 days of storage. Lipid oxidation is one main reason for the development of rancidity in roasted peanuts. Bazkiaee et al. [285] investigated the effect of different concentrations of edible coatings, including β-glucan and CS, on lipid oxidation. The β-glucan/CS coated peanuts show the least level in moisture contents, fungal counts, and mean total aflatoxin levels, thus the least lipid oxidation values. They find that the lipid oxidant is significantly reduced in β-glucan/CS-coated peanuts, further extending the shelf life of peanuts. So, they conclude that coating peanuts with β-glucan/CS coating films is a helpful method for storing peanuts.

Apart from cashews and peanuts, pistachios are another member of the nut family, which is rich in dietary fiber, minerals, and unsaturated fatty acids. Fresh pistachios are highly perishable due to their soft skin and, therefore, have a short shelf life after harvesting. To address this problem, numerous researchers are exploring the potential of CS-based nanofibers as edible coating films for preserving fresh pistachios. Mahdavi et al. [286] focused on the storage of pistachios and examined the effect of CS-based coating combined with cold plasma on the quality and safety of pistachios. Their research results show that 1.5% CS and 120 s of cold plasma could significantly reduce the amount of mold and yeast after 120 days of storage. They concluded that 1.5% CS and 120 s of cold plasma can preserve pistachios in the best way (*p* < 0.05), providing a new approach to preserving fresh pistachios. Similarly, focusing on fresh pistachios, Taghipour et al. [245] evaluated the effect of CS, nano-CS, and CS/ZnO on the quality and safety of fresh pistachios during storage. The results show that 250 and 500 mg·L^−1^ of CS/ZnO coatings are the most efficient methods for preserving the quality of fresh pistachios compared to the other two. In this way, farmers can significantly reduce hull browning and minimize the loss of fresh pistachios during harvest. Similar work performed by Taghipour et al. [241] examined the effect of CS (500 and 1000 mg·L^−1^), nano-CS (250 and 500 mg·L^−1^), and CS/TiO_2_ (250 and 500 mg·L^−1^) on the storage of fresh pistachios. They find that CS/TiO_2_ (250 and 500 mg·L^−1^) nanocomposites coating for fresh pistachios reduces microbial contamination, weight loss, phenylalanine ammonia-lyase activity, and saturated fatty acids and increases the unsaturated fatty acids, antioxidant properties, and shelf life of pistachios to 60 days. From the above two studies on fresh pistachios, we conclude that the effects of CS-based nanocomposites such as CS/ZnO and CS/TiO_2_ on the preservation of fresh pistachios are consistently better than those of pure CS and nano-CS.

These results suggest that CS-based nanofiber coating films have the potential to be used as packaging materials for nuts, including cashews, peanuts, and pistachios.

## 5. Modeling and Simulation Techniques and Insights

### 5.1. Models, Methods, and Mechanistic Insights

In parallel with experiments, MD simulation can track the molecular or atomic-level interactions of CS with other polymers in the food field. These computational modeling and simulations enable the prediction and provide explanations of experimentally observed macromolecular structures, dynamics, thermodynamics, and microscopic and macroscopic properties of CS-based polymers [287]. Compared to experimental work, one of the key advantages of simulations is their ability to provide atomistic or molecular level insights into phenomena that are difficult or even impossible to capture through experiments alone, such as the hydrogen bonding networks, chain entanglement, and diffusion pathway [288]. Moreover, simulations allow for systematic variation in parameters such as temperature, polymer concentration, and conformation in a controlled and cost-effective way, reducing the need for expensive experimental trials.

**Figure 3:**
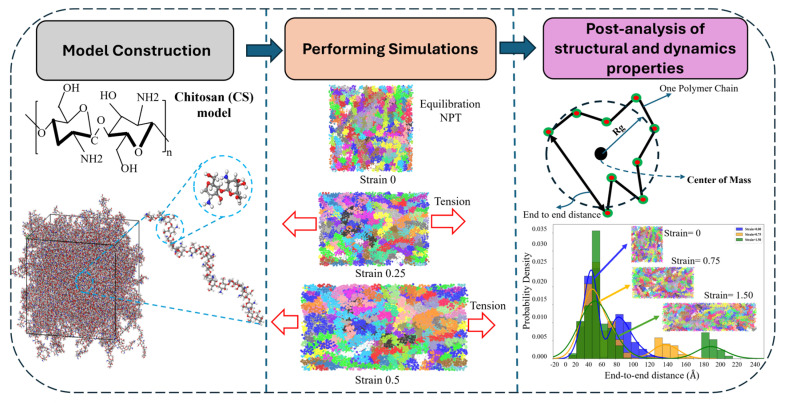
Schematic illustration of the key steps in polymer simulations, including model construction, performing simulations, and post-analysis of structural and dynamic properties.

The polymer simulation process typically involves three key stages: developing appropriate models, running polymer simulations, and analyzing the simulation results, as schematically represented in Figure 3. For classical molecular dynamics simulations, most models are generally categorized into two categories: atomistic models and CG models. The atomistic model presents each atom explicitly and provides insights into specific atomic-level interactions in known morphologies. Atomistic simulations allow for the investigation of local monomer-level fluctuations and monomer–monomer interactions [289,290]. In contrast, CG models simplify the system by grouping atoms into larger interaction sites or beads, enabling the simulations with reduced orders of freedom, and thus computational costs [291,292,293]. For both models, the appropriate force field—a mathematical description of interatomic or inter-bead interactions—is core to molecular dynamics simulations, which determines the accuracy of the simulation results. The common atomistic force fields include condensed-phase optimized potentials for atomistic simulation studies (COMPASS), polymer consistent force field (PCFF), and consistent-valence force field (CVFF), while CG models often utilize the MARTINI or tailor-made potentials developed through systematic coarse-graining techniques [294,295,296].

A critical step in simulations for polymers is the equilibration of the initial system. To expedite this process, it is usually achieved by mimicking the thermal annealing process, a common practice in material processing, where the system is gradually heated above the polymer’s melting point to allow the chains to relax and adopt thermodynamically favorable conformations. The temperature is then slowly reduced to ambient conditions, relieving internal stresses and enabling the system to reach equilibrium [297,298,299]. Once equilibration is achieved, various structural and dynamic properties can be calculated to characterize the polymer system. Commonly analyzed metrics include the radius of gyration [300,301,302,303], end-to-end distance [303,304], density [305,306], mean-squared displacement (MSD) [307,308,309], diffusion coefficient [309,310], radial distribution function (RDF) [311,312], and stress–strain responses [313,314,315].

**Figure 4:**
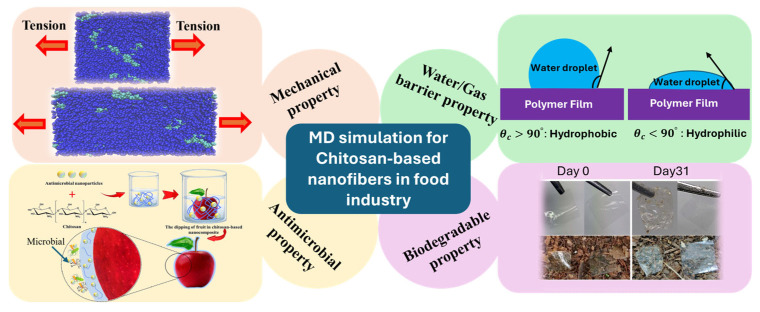
Four representative properties of CS-based nanofibers that can be investigated through computational simulations, including mechanical properties, water and gas barrier performance, antimicrobial activity, and biodegradability. Reprinted with permission from refs. [316,317]. Copyright MDPI, 2024; Elsevier, 2023.

Understanding the molecular mechanisms underlying the functional properties of CS-based polymers is essential for their optimized application in food packaging and the broader food industry. Such insights enable the rational design and optimization of materials tailored to specific requirements, including enhanced antimicrobial activity, improved gas and water barrier performance, and superior mechanical strength. Computational modeling and simulation act as a critical link between molecular-scale interactions and macroscopic properties. These tools allow researchers to investigate how atomistic and molecular-level phenomena—such as hydrogen bonding, chain entanglement, and nanoparticle dispersion—influence bulk behaviors like permeability, antimicrobial efficacy, and mechanical integrity. The following subsections summarize computational studies on mechanical, barrier, antimicrobial, and biodegradable properties of CS-based nanofibers and coating films relevant to food packaging applications. Figure 4 summarizes the key roles of MD simulations in evaluating CS-based nanofibers for food industry applications, focusing on their mechanical, water/gas barrier, antimicrobial and biodegradable properties. 

Importantly, parameters derived from MD simulation can inform the optimization of CS-based polymer systems and their performance. For example, in the work of Sanjib et al. [318], simulations of tension, compression, and shear with and without hydrogen bonding revealed that the absence of hydrogen bonding led to significant reductions in transverse tensile, compressive, and shear moduli (by 40–44%) and strengths (by 47–69%). These findings suggest that hydrogen bonding strength, as predicted by MD, correlates strongly with the experimentally observed mechanical performance of electrospun nanofiber films. Similarly, the MSD of polymer atoms calculated via MD provides insight into polymer chain mobility and plasticity—critical for developing flexible and durable packaging films. MSD analysis enables pre-screening of polymers to identify optimal blending partners for CS that improve ductility and reduce brittleness, thereby guiding material selection prior to costly experimental trials. In summary, MD simulations not only reveal fundamental molecular interactions but also serve as predictive tools for rational experimental design and performance enhancement in CS-based electrospun nanofibers.

### 5.2. Mechanical Properties

**Figure 5:**
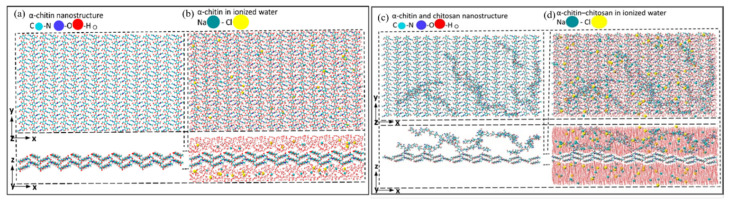
α-chitin nanostructure (**a**) without ionized water; (**b**) with ionized water in the *z*-direction. α-chitin–CS nanostructure (**c**) without ionized water; (**d**) with ionized water in the *z*-direction. Reprinted with permission from Ref. [319]. Copyright MDPI, 2023.

The mechanical properties of CS-based nanofibers—such as tensile strength, flexibility, durability, and lightweight—are critical for their applications in food packaging. To further extend applications of CS-based nanofibers or coating films, the mechanical performance of CS-based nanofibers should be enhanced and strengthened. Attaeyan et al. [167] performed MD simulations using LAMMPS on CS-silica aerogels reinforced with tricalcium phosphate (TCP) nanoparticles. They obtained stress and strain data from molecular dynamics simulations and calculated the stress–strain curve. In this work, they found that adding a small amount of TCP (1–3%) dramatically increases the ultimate strength (from 26.97 to 43.47 GPa) and Young’s modulus (from 681 to 1053 MPa) of nanocomposites. Above the 5% TCP added, the strength declined, indicating an optimal filler content. In numerous experimental works, researchers have made significant efforts to incorporate various nanoparticles or other materials such as metal oxides (e.g., ZnO, TiO_2_, CuO, CeO_2_), metal nanoparticles (e.g., Ag, Cu), and layered silicates such as montmorillonite (MMT) and halloysite nanotubes (HNTs) into CS-based nanofibers to enhance their mechanical performance. However, their atomic-level mechanism underlying the structure and performance of CS-based nanofibers remains largely unexplored. Atomistic MD has been used to quantify the relationship between CS structure and its mechanical properties. Salavati [319] built all-atomistic models of crystalline α-chitin sheets and chitin–CS hybrid immersed in water and applied uniaxial tensile loading in LAMMPS with the CHARMM36 force field. Figure 5a shows the atomic structure of the α-chitin nanostructures. And Figure 5b illustrates α-chitin immersed in ionized water at room temperature.

Figure 5 represents α-chitin–CS nanostructure with randomly distributed CS in the x-y plane along the z-axis, with and without ionized water [319]. The models in this work show that α-chitin has a higher directional elastic modulus of 51.76 GPa in the x-direction and 39.76 GPa in the y-direction than its CS bio composites. The larger mechanical stiffness of α-chitin can be attributed to its highly crystalline molecular structure, providing potential for food packaging applications that require load-bearing capabilities. Besides atomic simulation, CG MD has been used to study the chemically modified CS network. For example, Singhal et al. [320] built a CG model of acylated CS chains. This model predicts the formation of different network structures. Although this study focused on drug diffusion, the inferred network architecture also implies changes in mechanical behaviors, including the reduced overall stiffness, increased flexibility, and lower tensile strength of CS hydrogel. These simulation studies demonstrate that the structure and additives of CS-based nanofibers or films can be tailored. Designers of CS-based food packaging can utilize such MD/CG insights to strike a balance between strength and flexibility.

### 5.3. Gas and Water Barrier Properties

Water and gas barrier properties are the main factors in food packaging since moist and oxygen environments promote the growth of bacteria and accelerate food metabolism [321,322,323]. Zhao et al. [97] employed MD simulations to examine the intermolecular interactions between PCL and CS in electron-spun nanofiber films specifically designed for food packaging applications. The MD simulations in this work are performed using GROMACS 2021 with the Amber99SB force field under NPT ensemble conditions at 323.15 K and 1 bar. The simulation aimed to elucidate the structural evaluation and interaction dynamics, particularly the formation of hydrogen bonding, between PCL and CH molecules. Quantitative analysis of the root mean square deviation (RMSD) revealed a decrease in structural fluctuation for both polymers. The amount of hydrogen bonding between PCL and CS increases during the simulation process. This processive rise implies enhanced compatibility and interaction strength over time. Finally, they concluded that PCL/CS (2%) films, which have the best gas and water barrier properties, have the potential for use in fruit packaging applications. The results from the simulation work are consistent with their experimental work. In addition, Hudek et al. [324] utilized the fully atomistic MD simulations and enhanced sampling methods to study the absorption mechanisms of CS oligomers on a silica surface. The simulation results show that the calculated free energy of absorption of CS on a silica surface is 0.6 kcal/mol per monomer in 0.15 mol L^−1^aqueous solution. And the loading capacity of CS on a silica surface is about 0.094 mg m^−2^. These simulations have the potential to guide experimental design of CS-coated nanoparticles for food packaging applications.

### 5.4. Antimicrobial Efficacy

CS-based films are often used as food packaging materials, which can extend the shelf life of food products by inhibiting the growth of spoilage-causing microorganisms [317,325,326]. Fuster et al. [327] applied MD to CS-coated lipid bilayers to examine the role of polymer charge density—the simulated two CS aggregates with different total charges in the bilayers. In the low-charge system, CS penetrated the bilayer after 10 ns, causing local thinning and disruption, while highly charged CS spread out on the surface without inserting, leaving the membrane intact. These results show that optimal antimicrobial activity requires balanced charge density. They concluded that the surface charge density is a significant factor in membrane-disrupting efficacy. This work suggests that controlling CS’s protonation or substituting it could adjust its antimicrobial effectiveness while avoiding unnecessary film erosion during food packaging design. In addition, Arisoy and coworkers [328] combined experiments and atomistic coating to study the ampicillin-loaded CS-hyaluronic acid films as a model. From the MD simulation results, increasing HA concentration from 3% to 6% has no significant influence on ampicillin release about 32–33%, whereas 12% HA markedly increases the release, reaching 87% at 336 h. So, all-atomistic MD simulations revealed that a high HA fraction results in increased diffusion of ampicillin out of the matrix. In the context of food packaging, this work implies that blending CS with other biopolymers, including the polysaccharide-based biopolymers (alginate, starch, cellulose, and derivatives), protein-based biopolymers (gelatin, zein, and casein), and other natural polymers can be used to tailor the release rate of embedded antimicrobials.

In comparison with all-atomistic models, CG models group several atoms into a single bead, resulting in a significant reduction in the number of interaction sites. Besides the previous all-atomistic modes for CS-based nanofibers, Mohammed et al. [329] developed a CG model of DPPC liquid bilayers grafted with CS chains to simulate CS-coated films. They varied the grafting density of chains and identified three conformational regimes as coverage increases. In the dense brush regime, the long CS chains slowed down membrane lipid diffusion (the diffusion coefficient is reduced to $2.9\times 10^{-8}{\mathrm{cm}}^{2}/s$) and increased the overall bilayer stability (increased bilayer thickness to 4.1 nm). The simulation results in this work show that the thick CS layers, about 4.2 nm, make the membrane interface stiff. For food packaging films, this work suggests that coating density and chain length should be optimized. A dense CS coating would create a robust barrier to gas and water when the films are used in food packaging. Cambiaso et al. [330] recently introduced a new Martini 3 CG model for CS with tunable acetylation. This CG model can accurately capture the variations in the degree of acetylation and protonation states. In test simulations, CS showed a much stronger binding to anionic lipid monolayers than to zwitterionic ones, and the absorbed polymer increased the tensile strength. Having such a validated CG model enables future large-scale simulations of CS-based nanofibers. These simulation studies illustrate how molecular modeling can inform the design of antimicrobial food packaging.

### 5.5. Biodegradable Performance

Nowadays, many materials used in the food packaging field are petrochemical-based polymers due to their lower costs. However, petrochemical-based polymers are non-biodegradable, increasing environmental concerns regarding long-term pollution [331]. The most effective solution to this pollution problem is to produce bags or packaging for food based on biodegradable materials. CS is renowned for its biodegradable properties, making it and its biopolymer-based candidates for sustainable food packaging. In contrast to experiments, MD simulations help us understand the molecular mechanisms underlying biodegradability at the molecular and atomic levels. For example, the MD simulations have provided atomic insights into how enzymes degrade CS. Roman et al. [332] compared the structural and physicochemical properties of the human and egg-white lysozymes via a molecular docking approach. In this way, they obtained information concerning the specificity of the interactions between chito oligosaccharides with these enzymes. Finally, they concluded that the capacity of both lysozymes to cleave the CS is dependent on the polymer’s properties, which include MW, deacetylation, and pattern of deacetylation. This suggests that biodegradable efficacy is related to its structure. MD simulations have also demonstrated how the intrinsic structure and chemical modifications of CS affect its biodegradable properties. Using all-atomistic “de novo” MD self-assembly simulations, Romany et al. [333] concluded that acetylated monomers are likely to form strong intermolecular hydrogen bonds, which could explain the experimental observations that higher acetylation results in lower water solubility for CS.

In the practical food packaging field, less soluble CS means that CS-based nanofibers will biodegrade more slowly. In addition, the simulations conducted by Schopmans et al. [334] investigated three aminated CS polymers, where one, two, and three long-distance side chains are incorporated. The MD simulations revealed that the intermolecular H-bonds introduced by these side chains can significantly change chain conformation. The simulation also showed that CS with one and three aminated side chains have similar thermal decomposition and water content. In comparison, CS with two modified side chains tends to capture about 96 H-bonds more than CS with one or three amine side chains in the neutral forms. Chemical functionalization that attaches a specific group to the amino (-NH_2_) on the CS backbone offers a means to tune CS’s degradability. Such insights suggest that the functionality of CS-based materials can be tailored at a molecular level to meet the needs of specific applications in food packaging. These simulations highlight some specific molecular mechanisms that generally govern the biodegradation of CS in food packaging applications. A summary of recent MD simulation research on the applications of CS-based nanofibers in the food industry is provided in Table 5.

## 6. Key Challenges, Potential Solutions, and Future Perspectives

CS-based electrospun nanofibers present great potential for developing active and biodegradable food packaging due to the intrinsic bioactivity, structural tunability, and compatibility with functional additives. These systems can deliver multiple functionalities, including antimicrobial protection, moisture control, and gas barrier enhancement. However, the transition from laboratory prototypes to industrial-scale applications is hindered by several challenges. This section outlines critical obstacles and offers future directions centered on scalable fabrication, computational design, regulatory preparedness, and interdisciplinary innovation.

### 6.1. Scaling Electrospinning for Industrial Applications

As reviewed in Section 2 and Section 3, a variety of CS-based electrospun composite systems (such as CS-gelatin, CS-ZnO) demonstrate promising functions. However, most current studies are confined to laboratory-scale, single-needle electrospinning setups. These systems typically yield only a few milligrams to grams of fiber per hour [79], and it is far from sufficient for industrial packaging applications. Moving toward commercialization requires the development and standardization of high-throughput methods, such as needleless electrospinning, centrifugal spinning, or multi-nozzle electrospinning systems. These approaches, although technically feasible, must address issues like electric field interference, solvent recovery, environmental controls (e.g., humidity), and continuous substrate collection. Moreover, ensuring fiber uniformity, batch consistency, and active compound retention during large-scale production remains a persistent challenge.

For the material modifications and engineering strategies for high-throughput, low-energy production, CS derivatives or optimized solvent systems can reduce viscosity and promote jet stability. Incorporating auxiliary polymers like PVA or PEO enhances spinnability and enables multi-jet operation. Additionally, needleless setups such as rotating disk or drum systems have achieved significantly higher yields. One study reports nanofiber production rates up to ~15.9 g/hour using a rotary-disk setup—over two orders of magnitude higher than typical single-needle systems [350]. Furthermore, emerging techniques such as adopting electroblowing or bath-assisted electrospinning techniques to facilitate solvent evaporation and improve fiber collection with lower energy input [351]. These approaches demonstrate how materials innovation, combined with equipment engineering, can help overcome current limitations in electrospinning throughput and scalability.

The other important problem is the trade-off between the mechanical strength and biodegradability of CS. The biodegradation rate of CS is influenced by its DDA and crystallinity [352]. Highly deacetylated CS (DDA > 85%) tends to exhibit high crystallinity, making it more resistant to enzymatic degradation by lysozyme. In contrast, CS with a lower DDA (i.e., higher degree of acetylation) presents a more amorphous structure, which is more accessible to enzymatic attack and thus degrades more rapidly. However, this increase in biodegradability often comes at the cost of reduced mechanical strength. One promising strategy to balance these competing properties is polymer blending. For example, blending CS with other biodegradable polymers such as starch [353] or gelatin [354] can disrupt crystalline regions and improve degradation rates, while maintaining or even enhancing mechanical performance through intermolecular interactions or phase reinforcement. This approach provides a cost-effective and scalable solution for tuning both mechanical strength and environmental degradability in CS-based films.

### 6.2. Simulation-Driven and Machine Learning-Enabled Design

Computational modeling plays a vital role in the rational design of CS-based nanofiber systems. MD simulations, for instance, provide insight into polymer–filler compatibility, hydrogen bonding interactions, and the structural evolution of blended or crosslinked systems. These atomistic simulations can predict phase behavior, diffusion coefficients, and the release kinetics of encapsulated agents, significantly accelerating material development. However, MD simulations are limited to small-scale systems and cannot capture the macroscopic behavior of packaging films under mechanical stress. Finite element modeling (FEM) complements this by evaluating mechanical robustness, moisture barrier performance, and structural deformation under real-world loading conditions. Integrating MD and FEM can thus offer a multiscale understanding of material behavior.

Additionally, machine learning (ML) methods are emerging as tools to optimize electrospinning parameters. Trained on experimental data, ML models can predict fiber diameter, porosity, and release profiles based on variables such as voltage, polymer concentration, and flow rate [355,356]. Specifically, the support vector machines (SVM) and other models have been trained to correlate voltage, flow rate, humidity, etc., with fiber outcomes, achieving good accuracy in predicting fiber diameters [357]. In addition, ML models can be integrated into closed-loop feedback systems. For instance, real-time image capture or sensor input could monitor fiber characteristics during spinning, and AI algorithms could automatically adjust parameters (e.g., voltage or flow rate) to maintain consistent fiber quality. Such intelligent systems offer promising potential for stabilizing fiber morphology during large-scale production and are a key direction for future research and development. A future direction involves coupling MD, FEM, and ML into a unified digital workflow for the virtual prototyping of CS-based nanofiber systems tailored to specific food packaging requirements.

### 6.3. Regulatory and Safety Considerations

While CS itself is biocompatible and GRAS, the incorporation of nanomaterials (e.g., ZnO, TiO_2_, AgNPs) or bioactive compounds (e.g., EOs) introduces regulatory complexities, especially for direct food contact. Migration studies and toxicological assessments are essential to assess the safety and long-term behavior of these additives.

In addition, the nanoscale dimensions and high surface area of electrospun mats necessitate further evaluation of mechanical robustness, degradation behavior, and potential nanoparticle release over time. Regulatory bodies such as the FDA and European Food Safety Authority (EFSA) are increasingly scrutinizing nano-enabled materials in food packaging for their long-term biological safety. Taking the Ag as an example, the EFSA has set an acceptable migration limit for Ag at 0.05 mg/L in water and 0.05 mg/kg in food [153]. These thresholds serve as important benchmarks when evaluating the safety of CS-based packaging materials containing Ag-based nanofillers. To streamline translation to market, future research should include migration testing with food simulants, long-term stability assays, and compliance mapping with relevant food contact legislation (e.g., (EC) No. 1935/2004, (EC) No. 450/2009). Establishing harmonized safety protocols across the research community will support more transparent and efficient regulatory approval.

### 6.4. Toward Multiscale and Multifunctional Solutions

Future advancements will require interdisciplinary integration across polymer science, food engineering, materials processing, and data analytics. Hybrid systems that combine edible bioactive compounds (e.g., rosemary oil, green tea extract) with inorganic UV blockers or moisture absorbers can lead to “smart” packaging that simultaneously delays spoilage and monitors food quality. Similarly, stimuli-responsive CS nanofibers capable of releasing agents or changing color in response to pH or temperature shifts are promising candidates for next-generation intelligent packaging.

Moreover, exploring CS blends with other biopolymers (e.g. starch, collagen, zein) can fine-tune mechanical and barrier properties while maintaining biodegradability. Techniques such as coaxial electrospinning, 3D patterning, and surface functionalization will further expand design possibilities, enabling next-generation packaging materials that are responsive, sustainable, and multifunctional.

## 7. Conclusions

CS has emerged as a highly promising biopolymer for food packaging and preservation, owing to its inherent biocompatibility, biodegradability, antioxidant activity, and broad-spectrum antimicrobial properties. Its compatibility with various natural and synthetic polymers, as well as inorganic additives, allows for the fabrication of multifunctional edible films and coatings tailored to the specific needs of food preservation. With the growing demand for sustainable, safe, and active packaging solutions, CS-based materials have gained significant traction in both academic and industrial research.

Among the available fabrication techniques, electrospinning stands out as a versatile, scalable, and cost-effective method for producing CS-based nanofibers with tunable structures and enhanced functional properties. This review has provided a comprehensive overview of recent developments in polymer/CS and inorganic/CS electrospun nanofibers. The incorporation of synthetic polymers (e.g., PVA, PLA, PEO) and natural polymers (e.g., gelatin, starch, pectin), as well as inorganic nanomaterials (e.g., ZnO, AgNPs, MMT), has been shown to significantly improve the mechanical strength, thermal stability, barrier performance, antioxidant capacity, and antimicrobial/antiviral efficacy of CS-based nanofibers.

These nanofibers have demonstrated considerable potential across a wide range of food categories—including fruits, vegetables, dairy products, meats, seafood, and nuts—where their multifunctionality contributes to extending shelf life and reducing spoilage. Furthermore, emerging strategies such as coaxial electrospinning and incorporation of stimuli-responsive additives are paving the way for intelligent packaging systems capable of responding to environmental cues (e.g., pH, temperature) for real-time freshness monitoring and controlled release of active agents.

In parallel, MD and other computational modeling tools are beginning to play a pivotal role in the rational design of CS-based nanocomposites. These simulations provide fundamental insights into polymer–filler interactions, hydrogen bonding, molecular mobility, gas/moisture barrier formation, and antimicrobial agent release kinetics. The integration of modeling approaches such as MD, FEM, and ML enables a multiscale, data-driven framework for material optimization. Such simulation-guided design, when coupled with experimental validation, can accelerate the development of application-specific, high-performance nanofibers with improved biodegradability and minimal environmental impact.

Looking ahead, future research should prioritize the translation of laboratory-scale innovations into commercially viable, regulatory-compliant packaging systems. This includes the advancement of high-throughput electrospinning technologies, standardized safety assessments, and harmonized protocols for evaluating long-term stability and migration. Interdisciplinary collaboration—spanning polymer science, food engineering, materials processing, and data science—will be essential to unlock the full potential of CS-based electrospun nanofibers in next-generation food packaging.

In summary, this review has synthesized the current state of both experimental advancements and computational insights in CS-based electrospun nanofibers. It highlights their pivotal role in shaping the future of sustainable, smart, and multifunctional food packaging materials and outlines a forward-looking vision for their scalable and responsible implementation in the food industry.

## Figures and Tables

**Table 1:** Factors affecting the electrospinning process and resulting fiber morphology.

Factor Category	Specific Parameter	Typical Range	Effect on Electrospinning Process	Resulting Fiber Morphology	Refs.
Solution Parameters	Polymer Concentration/MW	7–10 wt% CS in acetic acid is considered high. Below ~3 wt%, fibers cannot form properly. CS (e.g., low Mw ~50–190 kDa vs. medium ~300–400 kDa vs. very high ~800+ kDa)	↑ concentration or MW→ ↑ viscosity and chain entanglement	The transition from beads to uniform fibers; ↑ fiber diameter	[70]
	Viscosity	100–3000 cP depending on the system	Too low→ beads. optimal→ uniform fibers; too high → jet blockage or ribbon fibers	Fiber diameter positively correlated with viscosity	[71]
	Surface Tension	30–50 mN/m	High tension → bead formation; Low tension → stable jet	Bead suppression with optimized tension	[72]
	Solution Conductivity	100–2000 μS/cm	↑ conductivity→ ↑ charge density and elongation	↓ fiber diameter; possible instability if excessive	[73]
Processing Variables	Needle Diameter	21–25 G (0.3–0.5 mm ID)	↑ diameter → thicker fibers due to larger initial jet	↑ fiber diameter	[74]
	Flow Rate	0.1–1.5 mL/h	Low → unstable cone; High → beads and thicker fibers	Optimal flow yields uniform thin fibers	[75]
	Applied Voltage	10–25 kV	↑ voltage → ↑ stretching force (to a limit)	↓ fiber diameter up to optimum; excessive → thicker fibers and beads	[76]
	Tip-to-Collector Distance (TCD)	10–25 cm	Short TCD → insufficient drying; Long TCD → weak stretching	Optimal distance yields thin, uniform fibers	[75]
Environmental Factors	Temperature	20–25 °C	↑ temperature → ↓ viscosity & ↑ evaporation	Often ↓ fiber diameter. excessive heating risks defects	[77]
	Humidity	30–50% RH	↑ RH → slower evaporation; possible porosity or bead formation	Fiber diameter decreases with moderate RH; excessive RH causes beads	[78]
	Air Flow	<0.5 m/s (laminar preferred)	Gentle airflow → enhanced elongation; Turbulent → instability	Thin, aligned fibers under controlled airflow	[79]

Note: ↑: increasing; ↓: decreasing; →: leading to.

**Table 3:** Natural polymers blended with CS.

Natural Polymer	Characteristics and Challenges	Notable Examples	Refs.
Sodium Alginate	Edible, biodegradable; blending with CS may cause precipitation, mitigated by pH control or coaxial spinning.	CS (5% *w*/*v* in acetic acid) and alginate (2% *w*/*v* in water) with PEO (4%). Electrospun mats with CS: alginate: PEO volume ratios of 20:80:100 or 80:20:100 for biomedical uses (e.g., drug-delivery/wound healing).	[108]
Starch	Poor spinnability alone; CS–starch blends enhance tensile and thermal properties via hydrogen bonding.	Blends of 7 wt% CS and 7 wt% starch (with 1% sorbitol); typical weight ratios: 85:15 or 70:30 (CS: starch); electrospun with PET for bio-based composites.	[109]
Gelatin	Enhances flexibility and adhesion; reduces viscosity/surface tension, aiding fiber formation.	30:70 (*w*/*w*) CS: gelatin blend yields uniform fibers with good cell compatibility; used in biomedical scaffolds.	[110]
Zein (Corn Protein)	Hydrophobic, lowers water vapor permeability (WVP); strengthens fibers via protein network formation.	Zein: PEO: CS = 87.5:10:2.5 (*w*/*w*); fibers loaded with α-tocopherol for antioxidant release in gastrointestinal patches.	[111]
Pectin	Edible, pH-responsive swelling; blending with CS often requires a mediator due to charge interactions.	CS: PVA and pectin: PVA blends (50:50 *w*/*w*); fibers exhibit antimicrobial activity for edible/active packaging films.	[112]

**Table 4:** Tensile properties of CS-based materials.

Composition	Tensile Strength (TS) (MPa)	Elongation at Break (EB) (%)	Relative Change (vs. Base)	Refs.
CS (electrospun film)—surrogate cast film	25.4 ± 2.1	32.9 ± 1.7	–	[105]
CS + CAP + ZnO NPs	97.6 ± 4.3	23.4 ± 1.1	TS ↑ ~285%, EB ↓ ~29% (vs. pure CS)	[105]
CS (cast film)	60.5 ± 3.1	13.3 ± 0.2	(base CS reference)	[198]
CS + TiO_2_ NPs	127 ± 8	13 ± 1	TS ↑ ~110%, EB ≈ same to slightly increased	[199]
CS (high stiffness/low elongation)	118.6 ± 5.5	6.1 ± 2.5	TS ↑ ~96%, EB ↓ ~54% (vs. cast CS)	[198]
CS + TiO_2_ NPs + EO	62.3 ± 3.7	2.9 ± 0.4	TS ↑ ~34%, EB ↓ ~78% (vs. cast CS)	[200]

Note: ↑: increasing; ↓: decreasing.

**Table 5:** Computational modeling and simulation studies of CS-based nanocomposites.

Material Systems	Insights from Simulations	Simulation Methods	Refs.
Carboxymethyl CS (CMCS)	Atomic-scale understanding of CMCS aggregation for tuning interactions for desired aggregation structures.	MD: GROMACS package. Force Field: standard GROMACS.	[45]
CS + ethylene oxide + zwitterion	Molecular-level mechanistic understanding of CS-based demulsification for oil/water systems, a versatile framework for the rational design of CS-based demulsification materials.	MD: GROMACS (2019.6). Force Field: GAFF; TIP3P for water molecules.	[335]
CS + Gellan	Mechanisms of aggregation and structure in Gellan-CS complexes for food applications	MD: GROMACS (2018). Force Field: CHARMM36 for CS. The newly developed force field [336] for Gellan.	[337]
2-hydroxypropyl-trimethylammonium chloride CS (HTCC) + amylose starch	Interfacial bonding mechanism between amylose, HTCC, and glutaraldehyde (GA), contributing to bacteriostatic performance, cytotoxicity, and transmittance.	MD: BIOVIA Materials Studio (MS) 2019. Interface modeling	[338]
CS + biodegradable polymers alginate (ALG)	Efficient absorbent design for dye removal of crystal violet (CRVT) and reactive black 5 (RBC 5) relevant to food safety	MD: MS 2021. Force Field: UFF. Optimization: $Dmol^{3}$ package.	[339]
Functionalized N-succinyl CS (NSC) + cinnamaldehyde	Molecular-level non-contact mechanisms of CIN-NSC antimicrobial preservation film.	Model: I-TASSER Online Server for *B. cinerea* CYP51 protein; AutoDock Vina 1.2.0 software for CYP51-CIN. MD: Amber20	[340]
CS + graphene oxide (GO-CS) + β-galactosidase	Stability and activity of β-galactosidase were enhanced upon interaction with CS and GO-CS nanocomposites, correlating positively with the increasing ratio of GO.	Model: RCSB database for PDB structure of β-galactosidase (sequence code 5MGC). PubChem for CS (Pubchem CID:71853) and graphene oxide (Pubchem CID:163320950) MD: GROMACS 2022.3 Force Field: OPLS-AA/L.	[341]
CS + bentonite	Complex interaction between the linuron molecule and the simulated bentonite-CS surface.	Model: polarized continuum model (PCM) of solution. Optimization: DFT method with the B3LYP functional and the 6–311+g(d,p) basis set.	[342]
CS + Cellulose nanofibrils (CNF) + Iron oxyhydroxide (FeOOH)	Confirm the stability and effectiveness of Se (IV) absorption at the molecular level.	Model: MS 2010. MD: LAMMPS Force Field: SPC/E parameters for H_2_O, previous studies [343,344] for HSeO_3_^−1^, OPLS force field for CNFs and CS.	[345]
carboxymethyl CS (CMCS) + CaCl_2_ + 3-PLA (antibacterial agent)	CaCl2 improves the O_2_ and H_2_O barrier properties of composite films at the molecular level.	Model: Amorphous Cell module in MS. Force Field: COMPASS	[346]
Glassy carbon electrode (GCE) + NiFe_2_O_4_ and CS	DFT calculations supported the electrochemical oxidation of ERT-B as an additive in food. MC simulations indicate strong stability and interactions between ERT-B and the sensing catalyst (NiFe_2_O_4_-CS).	Simulation: DFT + MC. Model: PUBCHEM for the 3D structure of ERT-B. PUBCHEM for CS. DFT calculations: DMol3 module in MS with the DNP basis set. MC simulations: Absorption Locator module in MS with UNIVERSAL force field.	[347]
CS + Tilapia surimi gels	Mechanisms and interactions between surimi MPs with CS under MW treatment.	Homology modeling: Swiss model by ChemDraw software (Cambridge Soft Co., Ltd., Cambridge, MA, USA) MD: GROMACS (Version 2019.6 GPU).	[348]
CS + Carrageenan multilayers	Devise efficient biocompatible macroion film formation.	Model: GROMACS MD: GROMACS 2016.4 package. Force Field: CHARMM36 for CS molecules; TIP3P for water.	[349]

## Data Availability

Data sharing is not applicable.

## Abbreviations

The following abbreviations are used in this manuscript:

CS	Chitosan
GRAS	Generally Recognized as Safe
FDA	U.S. Food and Drug Administration
EFSA	European Food Safety Authority
DFT	Density Functional Theory
MC	Monte Carlo
MD	Molecular Dynamics
CG	Coarse-Grained
FEM	Finite Element Modeling
ML	Machine Learning
XRD	X-ray Diffraction
AFM	Atomic Force Microscopy
FTIR	Fourier-transform infrared
UTS	Ultimate Tensile Strength
PVA	Polyvinyl alcohol
PEO	Polyethylene oxide
PCL	Polycaprolactone
PLA	Polylactic acid
PEG	Polyethylene glycol
PVP	polyvinylpyrrolidone
TFA	Trifluoroacetic acid
DCM	Dichloromethane
ZnO	Zinc Oxide
TiO_2_	Titanium Dioxide
CuO	Copper Oxide
CeO_2_	Cerium Oxide
MgO	Magnesium oxide
CaCO_3_	Calcium Carbonate
MMT	Montmorillonite
HNTs	Halloysite Nanotubes
GO	Graphene Oxide
CNF	Cellulose Nanofibrils
RMSD	Root Mean Square Deviation
MSD	Mean-Squared Displacement
TCP	Tricalcium Phosphate Nanoparticles
LAMMPS	Large-scale Atomic/Molecular Massively Parallel Simulator
PCFF	Polymer Consistent Force Field
CVFF	Consistent-Valence Forcefield
NMR	Nuclear Magnetic Resonance
COMPASS	Condensed-phase Optimized Potentials for Atomistic Simulation Studies
GCE	Glassy Carbon Electrode
BOA	Blood Orange Anthocyanins
*S. aureus*	*Staphylococcus aureus* and *Escherichia coli*
*E. coli*	*Escherichia coli*
